# Safe and Effective Treatment for Anemic Patients With Chronic Kidney Disease: An Updated Systematic Review and Meta-Analysis on Roxadustat

**DOI:** 10.3389/fphar.2021.658079

**Published:** 2021-07-02

**Authors:** Mei Tang, Changyu Zhu, Ting Yan, Yanglin Zhou, Qin Lv, Junlan Chuan

**Affiliations:** ^1^ Department of Pharmacy, Sichuan Academy of Medical Sciences and Sichuan Provincial People’s Hospital, Chengdu, China; ^2^ Personalized Drug Therapy Key Laboratory of Sichuan Province, School of Medicine, University of Electronic Science and Technology of China, Chengdu, China; ^3^ Department of Respiratory and Critical Care Medicine, Sichuan Academy of Medical Science and Sichuan Provincial People’s Hospital, School of Medicine, University of Electronic Science and Technology of China, Chengdu, China

**Keywords:** roxadustat, chronic kidney disease, renal anemia, safety, efficacy, meta-analysis

## Abstract

**Background:** Roxadustat is a new oral drug for anemia in chronic kidney disease (CKD). This study aimed to synthesize the evidence from randomized controlled trial (RCT)-based studies that estimated the efficacy and safety of roxadustat in anemia patients with non-dialysis-dependent (NDD) and dialysis-dependent (DD) CKD.

**Methods:** We searched the PubMed, Web of Science, and Cochrane Central Register of Controlled Trials (CENTRAL) databases for related published studies. Moreover, we manually searched relevant pharmaceutical company websites and two international clinical trial registers to search for published and unpublished RCTs comparing roxadustat with erythropoietin-stimulating agents (ESAs) or placebo.

**Results:** Fifteen RCTs (seven for DD-CKD patients, eight for NDD-CKD patients) were included in the meta-analysis, with 10,189 patients, 4,810 DD-CKD patients, and 5,379 NDD-CKD patients enrolled. Compared with ESAs (epoetin alfa or darbepoetin alfa) and placebo, roxadustat raised the hemoglobin level [weighted mean difference (WMD): 0.82 g/dL; 95% confidence interval (CI): 0.43–1.21], transferrin level (WMD: 0.5 g/L; 95% CI: 0.34–0.65), and TIBC level (WMD: 41.79 μg/dL; 95% CI: 38.67–44.92) and lowered the hepcidin level (WMD: −37.38 ng/ml; 95% CI: −46.63– −28.12) in both the DD-CKD and NDD-CKD patients with renal anemia. Roxadustat improved hemoglobin response and lowered the ferritin and TAST levels in the NDD-CKD patients but not in the DD-CKD patients. Furthermore, there was no difference between the treatment-emergent adverse events (TEAEs) of roxadustat and that of ESAs or placebo. But the incidence of serious TEAEs in the roxadustat group was significantly higher with NDD-CKD patients (OR: 1.15; 95% CI: 1.02–1.29).

**Conclusion:** This study confirmed that roxadustat therapy could alleviate the anemia of DD-CKD and NDD-CKD patients by raising the hemoglobin level and regulating iron metabolism, but increased serious incidences of treatment-emergent adverse events (TEAEs) in NDD-CKD patients.

## Introduction

Anemia remains one of the common complications of chronic kidney disease (CKD), which is due to the relative or absolute deficiency of erythropoietin (EPO), resulting in the inability to maintain normal red blood cell (RBC) levels (Babitt and Lin, 2012; Webster et al., 2017). The prevalence of anemia in CKD (also known as renal anemia) increases with the CKD stage, up to 53.4% at stage 5 in the United States (Stauffer and Fan, 2014). A cross-sectional study showed that the overall prevalence of CKD in China was 10.8%, making it as a serious public health problem (Zhang et al., 2012). Anemia occurred in 51.5% of the cases in all the stages of non-dialysis-dependent CKD (NDD-CKD), even up to 90.2% in stage 5 NDD-CKD in Shanghai, China (Li et al., 2016). In addition, renal anemia is an important risk factor of progression of CKD, increased incidence and mortality of cardiovascular complications, decreased quality of life (Drawz and Rahman, 2015). Maintaining anemia at an appropriate level is important for improving the life quality and survival rate of CKD patients.

Renal anemia is believed to be caused by EPO deficiency, functional iron deficiency, and other factors including blood loss or inflammation (Mcfarlane et al., 2008). Based on its etiologies, the most mature treatment currently used clinically is EPO and iron replacement therapy. The current guidelines on the management of anemia in CKD mainly focus on erythropoietin-stimulating agent (ESA) therapy, iron therapy, and blood transfusion (Cameron, 1999; Locatelli et al., 2004; Hainsworth, 2006; Kdoqi, 2007; Locatelli et al., 2013; Padhi et al., 2015). Effective iron supplementation can alleviate anemia and reduce the ESA dose or even help patients exempting from ESA treatment. In end-stage renal disease, especially in dialysis-dependent CKD (DD-CKD) patients, the effect of traditional oral iron is greatly weakened because intestinal iron absorption is limited. Intravenous iron supplementation has become the main way for patients to reach the targeted hemoglobin (Hb) level and to reduce the ESA usage (O’lone et al., 2019). However, the risk of developing cardiovascular complications and infections in patients receiving intravenous iron supplementation is much higher than that in patients posed by oral iron supplementation (Agarwal et al., 2015; Pandey et al., 2016). During the maintenance treatment of ESA therapy, there exists difficulty of dose adjustment to make Hb levels stable because a considerable part of patients show low responsiveness even receiving high-dose ESAs, let alone high-dose ESAs may increase the cardiovascular event risk Drueke et al. (2006), Singh et al. (2006), Pfeffer et al. (2009), Solomon et al. (2010) and mortality (Phrommintikul et al., 2007). Therefore, more effective and safe treatments are required to overcome the limitations of the existing therapeutic drugs.

Hypoxia-inducible factor-prolyl hydroxylase inhibitors (HIF-PHI), new oral agents for anemia treatment in CKD inhibiting the activity of hypoxia-inducible factor-prolyl hydroxylase (HIF-PHD), can simulate the human hypoxia state, stabilize the HIF pathway and thus stimulate endogenous EPO production, upregulate transferrin receptor expression, increase the iron uptake by proerythrocytes, and promote the maturation of erythrocytes repleting with Hb (Bonomini et al., 2016; Gupta and Wish, 2017). A number of HIF-PHIs are currently undergoing phase 2 or 3 clinical trials, such as roxadustat (FG-4592, ASP1517, AZD9941), daprodustat (GSK-1278863), molidustat (BAY 85-3934), vadadustat (AKB-6548, MT-6548), and enarodustat (JTZ-951). Among them, roxadustat is the world’s first orally administered small-molecule HIF-PHI, which has been approved by the National Medical Products Administration (NMPA) in China for the treatment of anemia in NDD-CKD Chen et al. (2019b) and DD-CKD Chen et al. (2019a) patients, and by the Pharmaceuticals and Medical Devices Agency (PMDA) in Japan for the treatment of anemia in NDD-CKD patients (Dhillon, 2019). Roxadustat has been proven to induce RBC production while maintaining the plasma EPO levels within or near the normal physiologic range in multiple subpopulations of CKD patients (including in the presence of inflammation), without extra intravenous iron supplementation (Besarab et al., 2015; Besarab et al., 2016; Yan and Xu, 2020).

During the last couple of years, several meta-analyses were conducted to estimate the efficacy and safety of roxadustat for renal anemia. However, only two phase III trials were enrolled in two of previously published meta-analyses (Liu et al., 2020; Zheng et al., 2020). Conclusions about the efficacy and safety of roxadustat were based on phase II trials exclusively in other meta-analyses (Zhong et al., 2018; Jia et al., 2019; Hu et al., 2020). In addition, the number of studies and sample size of patients included in above meta-analyses was relatively small, not exceeding 9 studies and 1,010 participants, which might have an impact on the strength of meta-analysis. In addition, the duration of the studies enrolled in previous meta-analyses was relatively short, with a maximum follow-up of 26 weeks. Recently, the results of five large phase 3 clinical trials Akizawa et al. (2020a), Coyne et al. (2021), Fishbane et al. (2021), Provenzano et al. (2021), Shutov et al. (2021) of roxadustat were released, so we updated the meta-analysis. Randomized controlled trials that assessed the efficacy (hemoglobin levels and response) and safety (treatment-emergent adverse events) of roxadustat in DD-CKD and NDD-CKD anemia patients comparing with ESAs or placebo were retriedved with an aim to generate a robust conclusion on the effect of and safety of roxadustat.

## Methods

To ensure the transparency and clarity of reporting of systematic reviews and meta-analyses, our study was conducted in accordance with the Preferred Reporting Items for Systematic reviews and Meta-Analyses (PRISMA) (online Supplementary Table S1, http://www.prisma-statement.org/).

### Search Strategy

We searched PubMed, Web of Science, and Cochrane Central Register of Controlled Trials (CENTRAL) for studies up to April 19th, 2021, with no language restrictions. We used both MeSH and the free-text terms “chronic kidney disease” and “roxadustat” (online Supplementary Table S2). Two international clinical trial registering websites (clinicaltrials.gov, clinicaltrialsregister.eu) and relevant pharmaceutical company websites (https ://www. fibrogen. com/our-expertise/publication/, https://astellasclinicalstudyresults.com/wecome.aspx, https://astrazenecagrouptrials.pharmacm.com/ST/Submission/Search) were reviewed exhaustively to retrieve pertinent unpublished studies.

### Inclusion and Exclusion Criteria

The inclusion criteria were: 1) randomized controlled trial only; 2) adult CKD patients diagnosed with renal anemia with or without dialysis, regardless of race; 3) roxadustat as a treatment compared with placebo or ESAs; and 4) reported Hb as an outcome with or without iron metabolism detection index and adverse events (AEs). Studies completely met all the above four criteria were included. Reviews, case reports, abstracts, letters, editorials, expert opinions, studies involving healthy individuals, and retrospective studies were excluded.

### Outcomes Measurement

The primary-outcomes for this meta-analysis were changes of hemoglobin (Hb; g/dL) levels from baseline to the study endpoint and Hb response. The secondary outcomes were the changes of iron metabolism biomarkers [hepcidin (ng/mL), transferrin (g/L), total iron-binding capacity (TIBC μg/dL), transferrin saturation (TSAT %), and ferritin (μg/L)], Hb changes in subjects with baseline C-reactive protein (CRP) > upper limit of normal (ULN), incidence of treatment-emergent adverse events (TEAEs), and incidence of serious TEAEs. Specifically, serious TEAEs included adverse events: result in death; are immediately life-threatning; require in-patient hospitalization or prolongation of existing hospitalization; jeopardize the patient or require medical intervention to prevent above outcomes.

### Data Collection and Quality Assessment

Two authors (T Yan and Y Zhou) independently screened the titles, abstracts or read the full text to determine the eligible studies according to the predefined inclusion and exclusion criteria. We designed a data extraction sheet containing the basic information of the enrolled studies (author, publication date, ClinicalTrials number), patient types, study phase, study type, interventions (dose, treatment duration, number), outcomes, and any therapy AEs. Two other authors (M Tang and C Zhu) extracted the data independently and discussed with each other over the difference. The risk of bias of each RCT was assessed using the Cochrane risk-of-bias tool. Five different types of bias were estimated for each study: selection bias (random sequence generation, allocation concealment), performance bias (blinding of participants and personnel), detection bias (blinding of outcome assessment), attrition bias (incomplete outcome data), and reporting bias (selective reporting). We also used the Grading of Recommendations Assessment, Development and Evaluation (GRADE) system to evaluate the quality of evidence for all outcomes (Guyatt et al., 2008). Disagreements over the study’s eligibility, data extraction, risk of bias or quality of evidence were consulted by the third person (J Chuan).

### Statistical Analysis

We performed all the analyses by Review Manager (RevMan, Version 5.3, The Nordic Cochrane Center, Cochrane Collaboration, Copenhagen, Denmark) and Stata 12.0. Heterogeneity between studies was evaluated using the Q statistic. *p* < 0.1 indicated a significant heterogeneity. When *p* ≥ 0.1, the fixed-effect model was adopted; If *p* < 0.1, heterogeneity was considered to exist between the included studies, thus the random-effect model was adopted. At the same time, subgroup analyses were conducted according to the patient type, intervention mode, and treatment duration to explore the potential source of heterogeneity. The potential publication bias was assessed by Begg’s and Egger’s tests with *p* < 0.1 indicating significant publication bias. When there was publication bias, the trim and fill test was used to analyze its impact on the results (Duval and Tweedie, 2000). Sensitivity analysis was conducted based on literature sources (excluding unpublished clinical trial data). The weighted mean difference (WMD) was used to evaluate the continuous outcomes (changes in the mean Hb, hepcidin, transferrin, TIBC, TSAT, and ferritin values) while the odds ratio (OR) was used to assess the dichotomous outcomes (Hb response, TEAEs, serious TEAEs), both parameters were represented by 95% confidence interval (Cl). All the tests were two-tailed, and statistical significance was set at *p* < 0.05.

## Results

### Fifteen Trials Were Finally Enrolled

**FIGURE 1:**
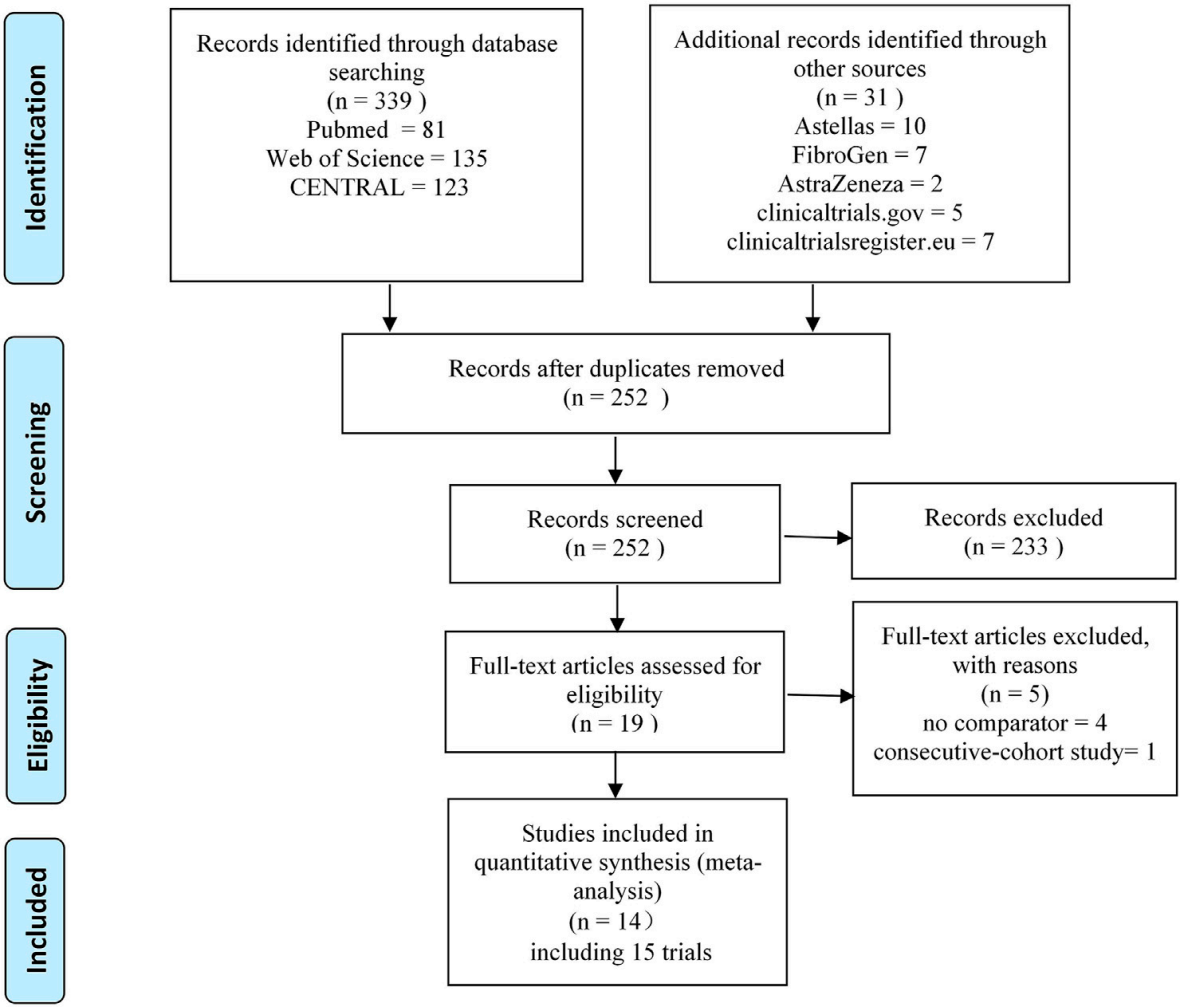
PRISMA flow diagram of eligible studies.

After removing the duplicates found from the screening of the titles and abstracts, 19 studies remained. Five of these were excluded after full-text review for the following reasons: no placebo or ESA comparator Besarab et al. (2016), Provenzano et al. (2016a), Akizawa et al. (2019b), Akizawa et al. (2020b) and consecutive cohort study (Provenzano et al., 2016b). Finally, 14 studies (including 15 trials) Besarab et al. (2015), Chen et al. (2017), NCT01888445 (2018), Akizawa et al. (2019a), Chen et al. (2019a), Chen et al. (2019b), NCT02278341 (2019), Akizawa et al. (2020a), NCT02174731 (2020), Coyne et al. (2021), Fishbane et al. (2021), NCT02021318 (2021), Provenzano et al. (2021), Shutov et al. (2021) were enrolled in this systematic review and meta-analysis. Of all the included trials, four trials with unpublished data were retrieved from the clinicaltrialsregister.eu NCT02278341 (2019), NCT02174731 (2020), clinicaltrials.gov (NCT02021318, 2021) and Astellas website (NCT01888445, 2018). The literature screening process that was employed is shown in Figure 1.

### Characteristics of Included Studies

All the enrolled studies were RCTs, including ten phase 3 trials Chen et al. (2019a), Chen et al. (2019b), NCT02278341 (2019), Akizawa et al. (2020a), NCT02174731 (2020), Coyne et al. (2021), Fishbane et al. (2021), NCT02021318 (2021), Provenzano et al. (2021), Shutov et al. (2021) and five phase 2 trials (Besarab et al., 2015; Chen et al., 2017; NCT01888445, 2018; Akizawa et al., 2019a) (Table 1). The total number of patients was 10,189: 4,810 DD-CKD patients (seven trials) and 5,379 NDD-CKD patients (eight trials). Of all the DD-CKD patient trials, four trials compared roxadustat with epoetin alfa Chen et al. (2017), Chen et al. (2019a), NCT02174731 (2020), Provenzano et al. (2021), two trials compared roxadustat with darbepoetin alfa NCT01888445 (2018), Akizawa et al. (2020a) and one trial compared roxadustat with both of above mentioned ESAs NCT02278341 (2019), and they were all not blinded except for two trials (NCT01888445, 2018; Akizawa et al., 2020a). In the NDD-CKD patient trials, placebos were used as control except in one trial NCT02021318 (2021), in which darbepoetin alfa was administrated as the comparator. The treatment duration ranged from 4 weeks to 4.5 years. The primary efficacy endpoint was mean hemoglobin change from baseline to the end of treatment duration and the proportion of patients achieving a hemoglobin response. All the trials allowed iron supplementation, using oral iron supplementation as preferred administration route and conditional intravenous iron supplementation.

**TABLE 1:** Characteristics of included trials.

Study name, year (clinical trials)	Duration of treatment	Phase of study, country (no. of centers)	Dosage of roxadustat	Study type	Type of patient	Baseline hemoglobin (Treatment/Comparison)	Iron supplement	Treatment (n)	Comparison groups (n)
Chen et al. (2019b) (NCT02652819)	26 weeks	Phase 3, China (30)	Initial dose: 70 mg (weight 40–60 kg) or 100 mg (weight ≥60 kg) TIW Dose adjustments (every 4 weeks): Maintain Hb level within 10.0 to 12.0 g/dL	Randomized, double-blind, placebo-controlled study	NDD-CKD	8.9 ± 0.8/8.9 ± 0.7	i.v. iron (Rescue therapy)	101	51 (placebo)
Chen et al. (2019a) (NCT02652806)	26 weeks	Phase 3, China (31)	Initial dose: 100 mg (weight 45–60 kg) or 120 mg (weight ≥60 kg) TIW Dose adjustments (every 4 weeks): Maintain Hb level within 10.0 to 12.0 g/dL	Randomized, open-label, epoetin alfa-controlled study	Stable DD-CKD	10.4 ± 0.7/10.5 ± 0.7	Allowed oral iron, i.v. iron (Rescue therapy)	204	100 (epoetin alfa)
NCT02174731 (2020)	4 years	Phase 3, worldwide (197)	Initial dose: 70 mg (weight 45–70 kg) or 100 mg (weight 71–160 kg) TIW Dose adjustments (every 4 weeks): Maintain an Hb of 11 ± 1 g/dL	Randomized, open-label, epoetin alfa-controlled study	Stable DD-CKD	NA	Encouraged oral iron, i.v. iron (Rescue therapy)	1,048	1,053 (epoetin alfa)
Fishbane (2021) (NCT02174627)	4 years	Phase 3, worldwide (385)	Initial dose: 70 mg TIW Dose adjustments (every 4 weeks): Maintain an Hb of 11 ± 1 g/dL	Randomized, double-blind, placebo-controlled study	NDD-CKD	9.1 ± 0.7/9.1 ± 0.7	Encouraged oral iron, i.v. iron (Rescue therapy)	1,393	1,388 (placebo)
NCT02278341 (2019)	52–104 weeks	Phase 3, worldwide (150)	Initial dose: 100 mg, 150 mg or 200 mg TIW Dose adjustments (every 4 weeks): Maintain Hb level within 10.0–12.0 g/dL	Randomized, open-label, ESA-epoetin alfa or darbepoetin alfa-controlled study	Stable DD-CKD	10.75 ± 0.62/10.77 ± 0.62	Encouraged oral iron, i.v. iron (Rescue therapy)	414	420 (epoetin alfa or darbepoetin alfa)
Shutov (2021) (NCT01887600)	52–104 weeks	Phase 3, European (125)	Initial dose: 70 mg (weight 45–70 kg) or 100 mg (weight 71–160 kg) TIW Dose adjustments (every 4 weeks): Maintain Hb level within 10.0–12.0 g/dL	Randomized, double-blind, placebo controlled study	NDD-CKD	9.078 ± 0.761/9.095 ± 0.721	Encouraged oral iron, i.v. iron (Rescue therapy)	391	203 (placebo)
Akizawa et al. (2020a) (NCT02952092)	24 weeks	Phase 3, Japan (58)	Initial dose: 70 mg or 100 mg TIW Dose adjustments (every 4 weeks): Maintain Hb level within 10.0–12.0 g/dL	Randomized, 2-arm parallel, double-blind, darbepoetin alfa-controlled study	Stable DD-CKD	11.02 ± 0.56/11.01 ± 0.60	Allowed oral iron, i.v. iron (Rescue therapy)	151	152 (darbepoetin alfa)
Provenzano et al. (2021) (NCT02052310)	52 weeks-4 years	Phase 3, worldwide (113)	Initial dose: 70 mg (weight <70 kg) or 100 mg (weight ≥70 kg) TIW	Randomized, open-label, epoetin alfa-controlled study	Incident DD-CKD	8.43 ± 1.044/8.46 ± 0.964	Encouraged oral iron, i.v. iron (Rescue therapy)	522	521 (epoetin alfa)
Akizawa et al. (2019a) (NCT01964196)	24 weeks	Phase 2, Japan (32)	Fixed-dose period (6 weeks): 50, 70, and 100 mg TIW Titration period (18 weeks): Maintain Hb at 10–12 g/dL	Randomized, double-blind Placebo-controlled study	NDD-CKD	9.4 ± 0.6/9.3 ± 0.7	Allowed oral iron	80	27 (placebo)
Chen et al. (2017) (NCT01599507)	12 weeks	Phase 2, China (13)	Low doses: 1.1–1.75 mg/kg High doses: 1.50–2.25 mg/kg TIW	Randomized, double-blind Placebo-controlled, sequential group, dose ranging study	NDD-CKD	8.8 ± 0.9/8.9 ± 0.8	Allowed oral iron, i.v. iron (Rescue therapy)	61	30 (placebo)
Chen et al. (2017) (NCT01596855)	7 weeks	Phase 2, China (10)	Low doses: 1.1–1.8 mg/kg Medium doses: 1.5–2.3 mg/kg High doses: 1.7–2.3 mg/kg TIW	Randomized, open-label active-comparator (epoetin alfa) dose-ranging study	Stable DD-CKD	10.7 ± 0.8/10.6 ± 1.0	Allowed oral iron, i.v. iron (Rescue therapy)	74	22 (epoetin alfa)
Besarab et al. (2015) (NCT00761657)	4 weeks	Phase 2a, United States (29)	0.7 g, 1.0, 1.5, and 2.0 mg/kg at BIW or TIW	Randomized, single-blind (subjects), placebo-controlled, sequential-group, dose-escalating study	NDD-CKD	10.3 ± 0.9/10.3 ± 0.9	Allowed oral iron, prohibited i.v. iron during the treatment	88	28 (placebo)
NCT01888445 (2018)	24 weeks	Phase 2, Japan (28)	Fixed dose period (6 Weeks): 50, 70 and 100 mg Titration period (18 weeks) TIW	Randomized, 4-arm parallel, double-blind (arms 1-3), open-label (arm 4), active-comparator study	Stable DD-CKD	8.84 ± 0.47/8.80 ± 0.51	NA	97	32 (darbepoetin alfa)
Coyne et al. (2021) (NCT01750190)	4.5 years	Phase 3, worldwide (163)	Initial dose: 70 mg (weight 45–70 kg) or 100 mg (weight 71–160 kg) TIW Dose adjustments (every 4 weeks): Maintain Hb level within 10.0–12.0 g/dL	Randomized, double-blind Placebo-controlled study	NDD-CKD	9.10 ± 0.75/9.09 ± 0.69	Encouraged oral iron, i.v. iron (Rescue therapy)	616	306 (placebo)
NCT02021318 (2021)	104 weeks	Phase 3, worldwide (125)	Initial dose: 70 mg (weight 45–70 kg) or 100 mg (weight 71–160 kg) TIW Dose adjustments (every 4 weeks): Maintain Hb level within 10.0–12.0 g/dL	Randomized, open-label, darbepoetin alfa-controlled study	NDD-CKD	NA	Encouraged oral iron, i.v. iron (Rescue therapy)	323	293 (darbepoetin alfa)

Notes: TIW, three times weekly; BIW, two times weekly; DD-CKD, dialysis-dependent chronic kidney disease; NDD-CKD, non-dialysis-dependent chronic kidney disease; Hb, hemoglobin; NA, not available.

### Quality Assessment of Included Studies and Evidence for all Outcomes

**FIGURE 2:**
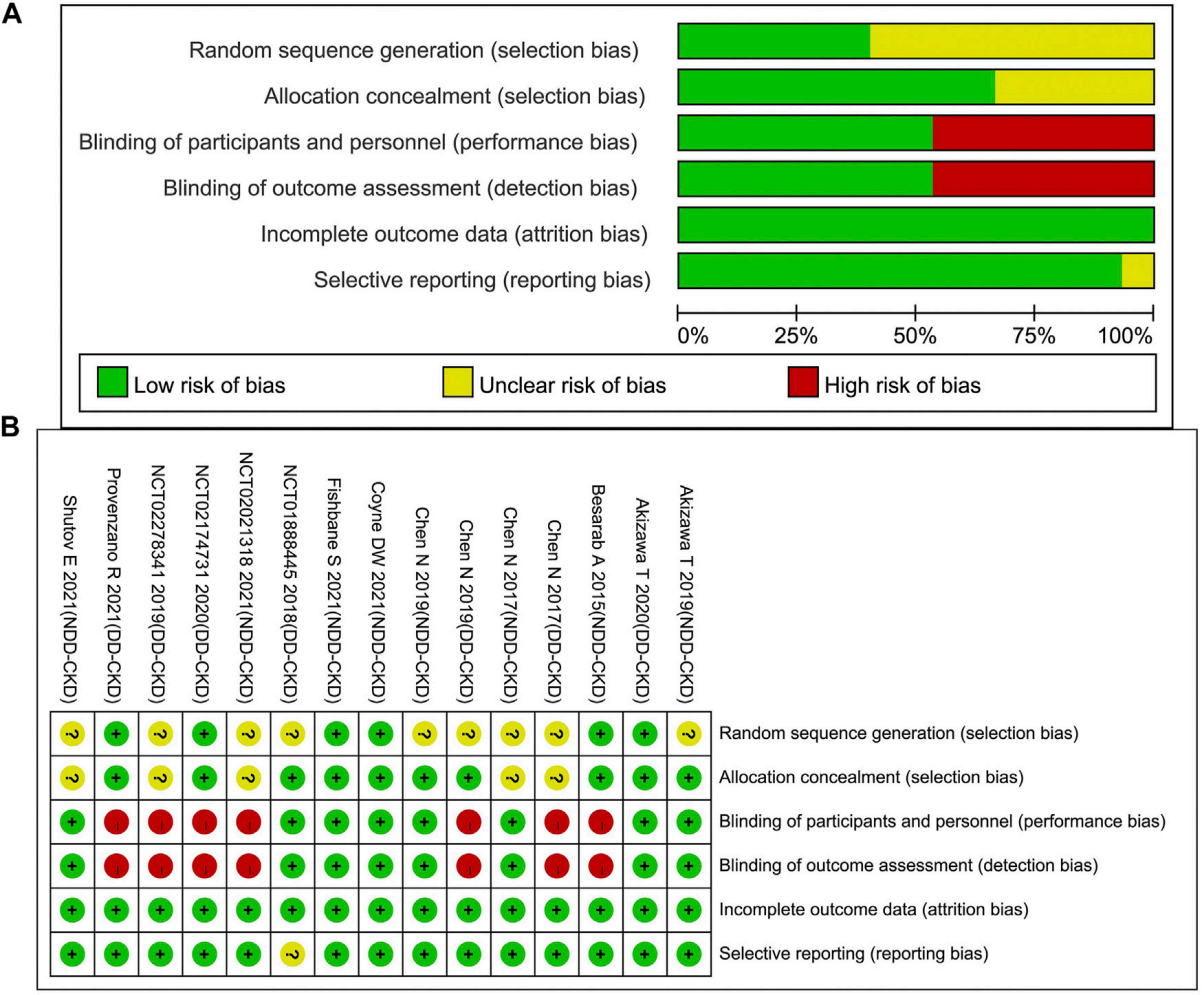
Risk of bias graph **(A)** and summary **(B)**.

The evaluation of the included RCTs through Cochrane Collaboration’s tool showed that high risk of bias mainly concentrated in DD-CKD patient trials, involving the blinding of the participants, personnel and outcome assessment (for open-label study) (Chen et al., 2017; Chen et al., 2019a; NCT02278341, 2019; NCT02174731, 2020; Provenzano et al., 2021). Defects in random sequence generation and allocation concealment also introduced some unclear risk of bias. The incomplete outcome data biases were assessed as low risk for all the studies because all the dropped-out patients were reported. The detailed assessment results are shown in Table 2 and Figure 2. We evaluated the quality of evidence for all outcomes, and found that the quality of evidence was inconsistent between the DD-CKD patients and NDD-CKD patients (online Supplementary Table S3). In NDD-CKD patients, the Hb and Hb response evidence evaluation results were low quality, which means that further research may have a great impact on the current results of roxadustat, and is likely to change the estimate. In DD-CKD patients, the Hb and Hb response evidence evaluation results were very low quality, which means that the credibility of the evidence results was very limited. The quality of the evidence for other outcomes was shown in the online Supplementary Table S3.

**TABLE 2:** The risk of bias of included trials (Authors judgment/Evidence for judgment).

Study	Random sequence generation	Allocation concealment	Blinding of participants and personnel	Blinding of outcome assessment	Incomplete outcome data	Selective reporting
Chen et al. (2019b) (NCT02652819)	Unclear risk/Not described	Low risk/Randomization was performed centrally and was stratified according to the use or nonuse of an ESA and the estimated GFR	Low risk/Double blind study	Low risk/Double blind study	Low risk/All dropped-out patients have been reported	Low risk/All relevant outcomes described
Chen et al. (2019a) (NCT02652806)	Unclear risk/Not described	Low risk/Randomized centrally in sequence, stratified according to the epoetin alfa baseline dose and dialysis method	High risk/Open-label study	High risk/Open-label study	Low risk/All dropped-out patients have been reported	Low risk/All relevant outcomes described
NCT02174731 (2020)	Low risk/Assign eligible patient unique randomization code through the IWRS/IVRS	Low risk/Automated randomization and treatment assignments will be provided by an IWRS/IVRS	High risk/Open-label study	High risk/Open-label study	Low risk/All dropped-out patients have been reported	Low risk/Based on the registered protocol in https://clinicaltrials.gov/ProvidedDocs/31/NCT02174731/Prot_000.pdf, all outcomes of the protocol were described
Fishbane et al. (2021) (NCT02174627)	Low risk/Assign eligible patient unique randomization code through the IVRS/IWRS	Low risk/Automated randomization and treatment assignments will be provided by an IWRS/IVRS	Low risk/Double blind study	Low risk/Double blind study	Low risk/All dropped-out patients have been reported	Low risk/Based on the registered protocol in https://clinicaltrials.gov/ProvidedDocs/27/NCT02174627/Prot_000.pdf, all outcomes of the protocol were described
NCT02278341 (2019)	Unclear risk/Not described	Unclear risk/Not described	High risk/Open-label study	High risk/Open-label study	Low risk/All dropped-out patients have been reported	Low risk/Information for this study available from Clincialtrials.gov outcomes reported as planned in Clinicaltrials.gov
Shutov et al. (2021) (NCT01887600)	Unclear risk/Not described	Unclear risk/Not described	Low risk/Double blind study	Low risk/Double blind study	Low risk/All dropped-out patients have been reported	Low risk/Information for this study available from Clincialtrials.gov, outcomes reported as planned in Clinicaltrials.gov
Akizawa et al. (2020b) (NCT02952092)	Low risk/By a web-based randomization system (EPS corporation, tokyo, Japan)	Low risk/Dynamic allocation was conducted using a biased-coin minimization approach	Low risk/Double blind study	Low risk/Double blind study	Low risk/All dropped-out patients have been reported	Low risk/Information for this study available from Clincialtrials.gov, outcomes reported as planned in Clinicaltrials.gov
Provenzano et al. (2021) (NCT02052310)	Low risk/Automated randomization and treatment assignments were provided by IXRS	Low risk/Automated randomization and treatment assignments were provided by IXRS	High risk/Open-label study	High risk/Open-label study	Low risk/All dropped-out patients have been reported	Low risk/Information for this study available from Clincialtrials.gov, outcomes reported as planned in Clinicaltrials.gov
Akizawa et al. (2019b) (NCT01964196)	Unclear risk/Not described	Low risk/Dynamic allocation was conducted using a biased-coin minimization approach	Low risk/Double blind study	Low risk/Double blind study	Low risk/All dropped-out patients have been reported	Low risk/All relevant outcomes described
Chen et al. (2017) (NCT01596855)	Unclear risk/Not described	Unclear risk/Not described	High risk/Open-label study	High risk/Open-label study	Low risk/All dropped-out patients have been reported	Low risk/All relevant outcomes described
Besarab et al. (2015) (NCT00761657)	Low risk/Treatment was assigned according to a randomization code provided by the statistical CRO/IVRS vendor	Low risk/Study drug was not dispensed in containers identifiable by subject as containing active or placebo capsules	High risk/Single blind Study (participant)	High risk/Single blind study (participant)	Low risk/All dropped-out patients have been reported	Low risk/All relevant outcomes described
NCT01888445 (2018)	Unclear risk/Not described	Low risk/Treatment arms notified by the web registration system	Low risk/Double blind study	Low risk/Double blind study	Low risk/All dropped-out patients have been reported	Unclear risk/Information for this study available from Clinicaltrials.gov, some secondary outcomes relevant for the present meta-analysis were not reported
Coyne et al. 2021 (NCT01750190)	Low risk/Automated randomization and treatment assignments were provided by IWRS	Low risk/Automated randomization and treatment assignments were provided by IWRS	Low risk/Double blind study	Low risk/Double blind study	Low risk/All dropped-out patients have been reported	Low risk/All relevant outcomes described
NCT02021318 (2021)	Unclear risk/Not described	Unclear risk/Not described	High risk/Open-label study	High risk/Open-label study	Low risk/All dropped-out patients have been reported	Low risk/All relevant outcomes described

Notes: ESA, Erythropoiesis Stimulating Agent; GFR, glomerular filtration rate; IWRS/IVRS, Interactive Web Response System/Interactive Voice Response System; IXRS, Interactive Voice and Web Response System; CRO, contract research organization.

### Meta-Analyses

#### Comparison of Effects on Hemoglobin

##### Hemoglobin - Roxadustat Increased the Level of Hemoglobin of Both Patients Groups

**FIGURE 3:**
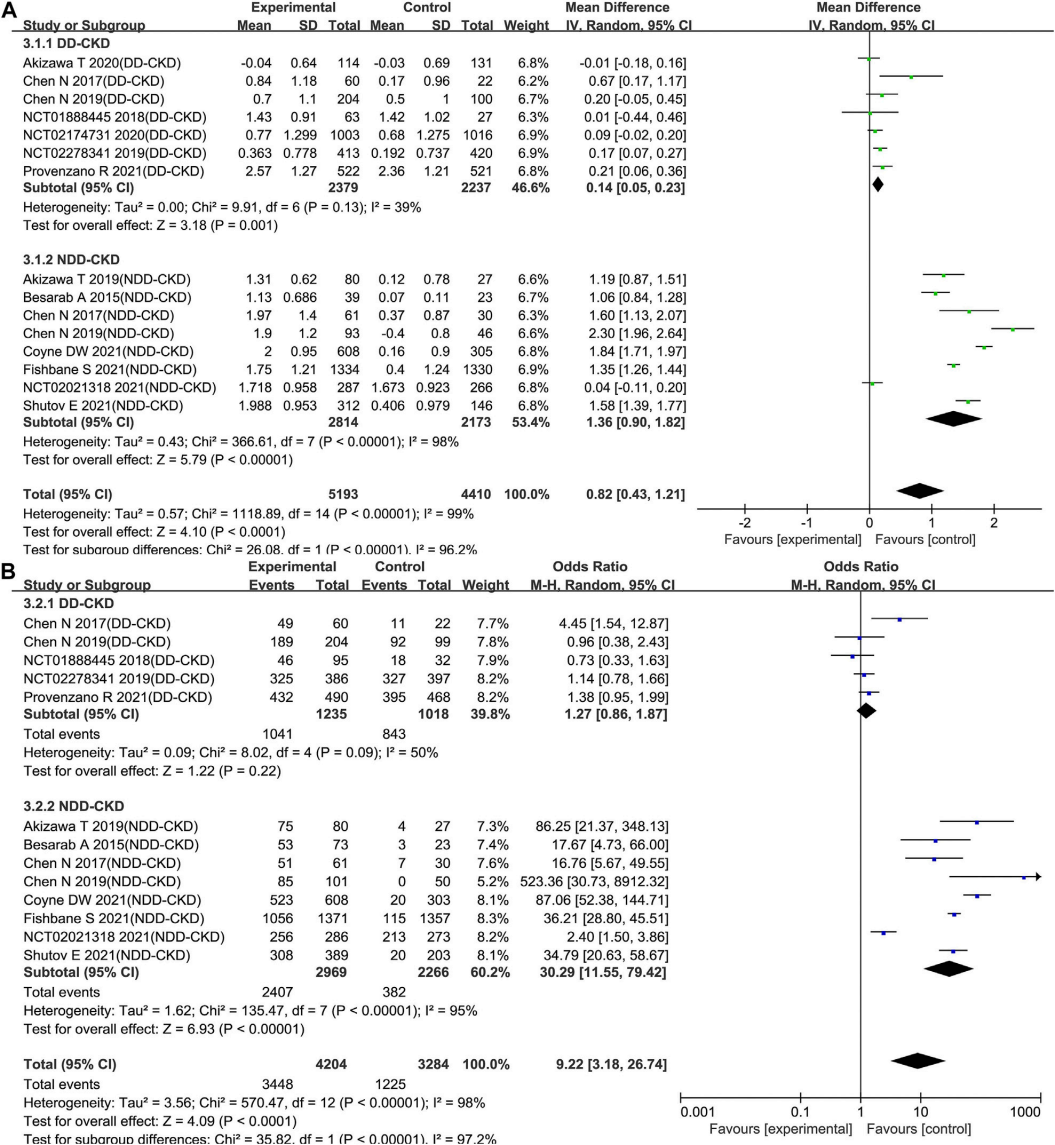
Effect of roxadustat compared with ESA or placebo on Hb level **(A)** and Hb response **(B)** in CKD patients. Notes: Hb, hemoglobin; CKD, chronic kidney disease; DD-CKD, dialysis-dependent chronic kidney disease; NDD-CKD, non-dialysis-dependent chronic kidney disease.

All the enrolled studies described changes of the Hb levels. Overall, the pooled results showed a significant rise in the Hb level in the roxadustat group compared to the ESAs or placebo groups (WMD: 0.82; 95% CI: 0.43–1.21; *p* < 0.0001; Figure 3A and Table 3). As the *p*-value of the heterogeneity test was 0.00001, subgroup analysis was conducted on the DD-CKD and NDD-CKD patients to analyze the causes of the heterogeneity.

**TABLE 3:** Summarize the results of roxadustat meta-analysis.

Outcomes	Group and subgroups	Number of studies	Number of patients	*Q* test *p* value	WMD/OR (95%CI)	*p* value
Hb (g/dL)	Overall	15	9,603	<0.00001	0.82 (0.43, 1.21)	<0.0001
	DD-CKD	7	4,616	0.13	0.14 (0.05, 0.23)	0.001
	NDD-CKD	8	4,987	<0.00001	1.36 (0.90, 1.82)	<0.00001
Hb response (%)	Overall	13	7,488	<0.00001	9.22 (3.18, 26.74)	<0.0001
	DD-CKD	5	2,253	0.09	1.27 (0.86, 1.87)	0.22
	NDD-CKD	8	5,235	<0.00001	30.29 (11.55, 79.42)	<0.00001
Hepcidin (ng/mL)	Overall	11	4,327	<0.00001	−37.38 (−46.63, −28.12)	<0.00001
	DD-CKD	4	1,722	0.32	−11.49 (−14.58, −8.41)	<0.00001
	NDD-CKD	7	2,605	<0.00001	−51.31 (−67.88, −34.74)	<0.00001
Transferrin (g/L)	Overall	6	963	<0.00001	0.50 (0.34, 0.65)	<0.00001
	DD-CKD	3	637	0.05	0.40 (0.30, 0.50)	<0.00001
	NDD-CKD	3	326	<0.00001	0.60 (0.24, 0.95)	0.0009
TIBC (μg/dL)	Overall	9	2,454	<0.00001	41.79 (38.67, 44.92)	<0.00001
	DD-CKD	4	1,382	0.0007	43.65 (33.78, 53.53)	<0.00001
	NDD-CKD	5	1,072	<0.00001	59.90 (38.85, 80.96)	<0.00001
TSAT (%)	Overall	12	4,004	<0.00001	−0.63 (−1.09, −0.16)	0.009
	DD-CKD	5	2,048	0.35	−0.35 (−1.06, 0.36)	0.34
	NDD-CKD	7	1,956	<0.0001	−2.84 (−5.03, −0.64)	0.01
Ferritin (μg/L)	Overall	11	3,532	<0.00001	−42.44 (−55.06, −29.82)	<0.00001
	DD-CKD	5	2,053	<0.00001	−33.64 (−83.39, 16.11)	0.19
	NDD-CKD	6	1,469	<0.00001	−54.01 (−76.90, −31.12)	<0.00001
Hb (g/dL) (CRP > ULN)	Overall	6	1,736	<0.00001	0.63 (0.09,1.17)	0.02
	DD-CKD	4	1,088	0.02	0.14 (−0.12, 0.40)	0.30
	NDD-CKD	2	648	<0.00001	1.47 (0.78, 2.17)	<0.0001
TEAEs	DD-CKD	6	2,704	0.49	1.21 (0.98,1.48)	0.08
	NDD-CKD	8	5,353	0.49	1.12 (0.95, 1.33)	0.18
Serious TEAEs	DD-CKD	6	4,709	0.39	1.12 (0.99, 1.26)	0.07
	NDD-CKD	8	5,353	0.99	1.15 (1.02,1.29)	0.02

Notes: NDD-CKD, non-dialysis-dependent chronic kidney disease; DD-CKD, dialysis-dependent chronic kidney disease; Hb, Hemoglobin; TIBC, total iron-binding capacity; CRP, C-reactive protein; ULN, upper limit of normal; TSAT, transferrin saturation; TEAEs, treatment-emergent adverse events.

The pooled results from all the DD-CKD patients showed that roxadustat significantly raised the Hb level compared with the ESAs (epoetin alfa and darbepoetin alfa) (WMD: 0.14; 95% CI: 0.05–0.23; *p* = 0.001; without heterogeneity [*p* = 0.13]; Figure 3A, Table 3). In the NDD-CKD patient subgroup, the Hb level significantly rose after roxadustat treatment than that after placebo and darbepoetin alfa treatment (WMD: 1.36; 95% CI: 0.90–1.82; *p* < 0.00001; with heterogeneity [*p* < 0.00001]; Figure 3A and Table 3).

In addition, we found that some of the Hb levels were presented as mean values while others were presented as least squares mean values. When the mean and LSM values were separated, the pooled results also showed that roxadustat significantly raised the Hb level both in the DD-CKD patients (online Supplementary Figure S1A) and in the NDD-CKD patients (online Supplementary Figure S1B).

##### Hemoglobin Response - Roxadustat Improved the Hemoglobin Response in Non-Dialysis-Dependent Chronic Kidney Disease Patients

Hb response was defined as a change from baseline Hb of ≥1 g/dL (baseline Hb > 8.0 g/dL) or ≥2.0 g/dL (baseline Hb ≤ 8.0 g/dL). Hb response data were extractable from thirteen trials Besarab et al. (2015), Chen et al. (2017), NCT01888445 (2018), Akizawa et al. (2019a), Chen et al. (2019a), Chen et al. (2019b), NCT02278341 (2019), Coyne et al. (2021), Fishbane et al. (2021), NCT02021318 (2021), Provenzano et al. (2021), Shutov et al., (2021) (five trials for DD-CKD patients, eight trials for NDD-CKD patients) with 7,488 participants enrolled. Subgroup analysis revealed that there was no significant difference about Hb response between roxadustat and ESAs in the DD-CKD patient group (OR: 1.27; 95% CI: 0.86–1.87; *p* = 0.22; Figure 3B and Table 3). However, in the NDD-CKD patient group, roxadustat improved the Hb response compared with placebo and darbepoetin alfa (OR: 30.29; 95% CI: 11.55–79.42; *p* < 0.00001; Figure 3B and Table 3).

#### Comparison of Effects on iron Utilization Parameters

**FIGURE 4:**
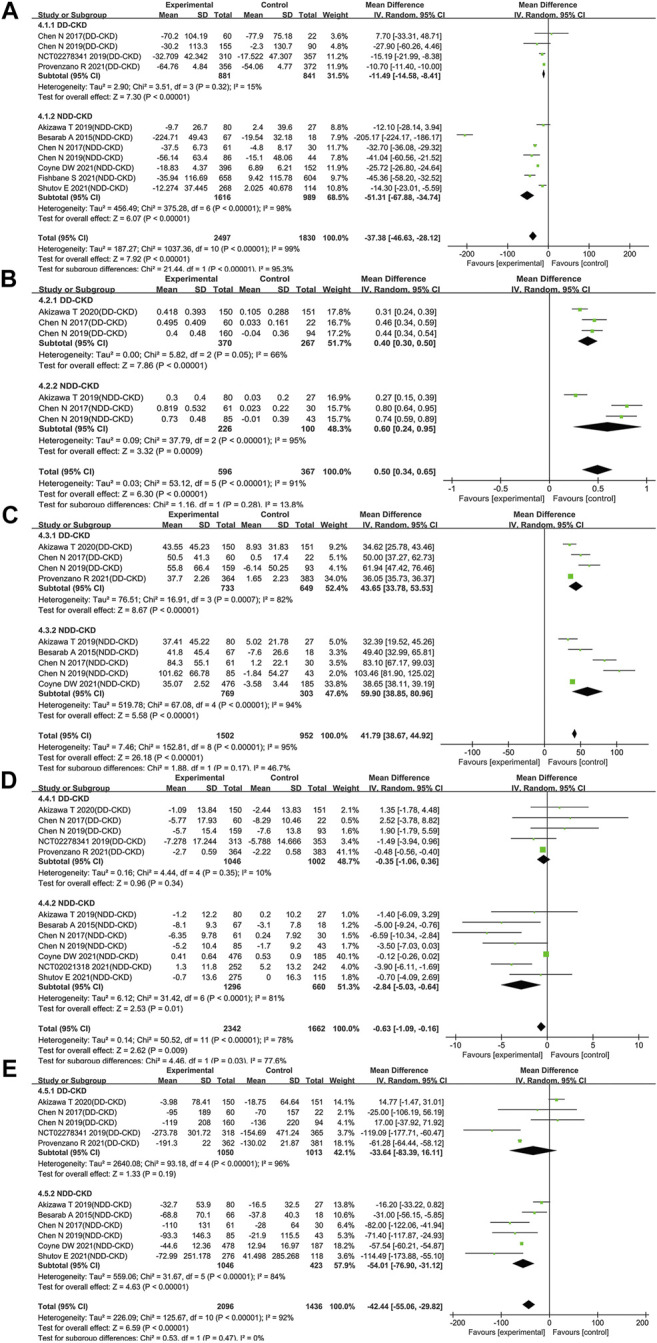
Effect of roxadustat compared with ESA or placebo on iron utilization parameters in CKD patients. Iron utilization parameters included hepcidin **(A)** transferrin **(B)** TIBC **(C)** TSAT **(D)** and ferritin **(E)**. Notes: CKD, chronic kidney disease; DD-CKD, dialysis-dependent chronic kidney disease; NDD-CKD, non-dialysis-dependent chronic kidney disease; TIBC, total iron-binding capacity; TSAT, transferrin saturation.

The improved ability of iron utilization is beneficial to renal anemia patients, so we investigated iron utilization related parameters including levels of hepcidin, transferrin, TIBC, TSAT and ferritin. A significant decrease of hepcidin was found in the roxadustat group as compared with the ESAs or placebo group (WMD: −37.38; 95% CI: −46.63 to −28.12; *p* < 0.00001; Figure 4A and Table 3). Moreover, transferrin and TIBC were significantly increased after treatment of roxadustat in both DD-CKD patient group (for transferrin: WMD: 0.40; 95% CI: 0.30–0.50; *p* < 0.00001; for TIBC: WMD: 43.65; 95% CI: 33.78–53.53; *p* < 0.00001; Figures 4B,C and Table 3) and in NDD-CKD patient group (for transferrin: WMD: 0.60; 95% CI: 0.24–0.95; *p* = 0.0009; for TIBC: WMD: 59.90; 95% CI: 38.85–80.96; *p* < 0.00001; Figures 4B,C and Table 3). However, roxadustat decreased the levels of TSAT and ferritin only in NDD-CKD patient group (for TSAT: WMD: −2.84; 95% CI: −5.03 to −0.64; *p* = 0.01; for ferritin: WMD: −54.01; 95% CI: −76.90 to −31.12; *p* < 0.00001; Figures 4D,E and Table 3) but not DD-CKD patient group (for TSAT: WMD: −0.35; 95% CI: −1.06 to 0.36; *p* = 0.34; for ferritin: WMD: −33.64; 95% CI: −83.39 to 16.11; *p* = 0.19; Figures 4D,E and Table 3). The suppression of hepcidin, reduction of TSAT and ferritin, increase of transferrin and TIBC indicated enhanced iron utilization by roxadustat especially in NDD-CKD patients.

#### Roxadustat Increased the Level of Hemoglobin in Non-Dialysis-Dependent Chronic Kidney Disease Patients With Elevated C-reactive Protein Levels

**FIGURE 5:**
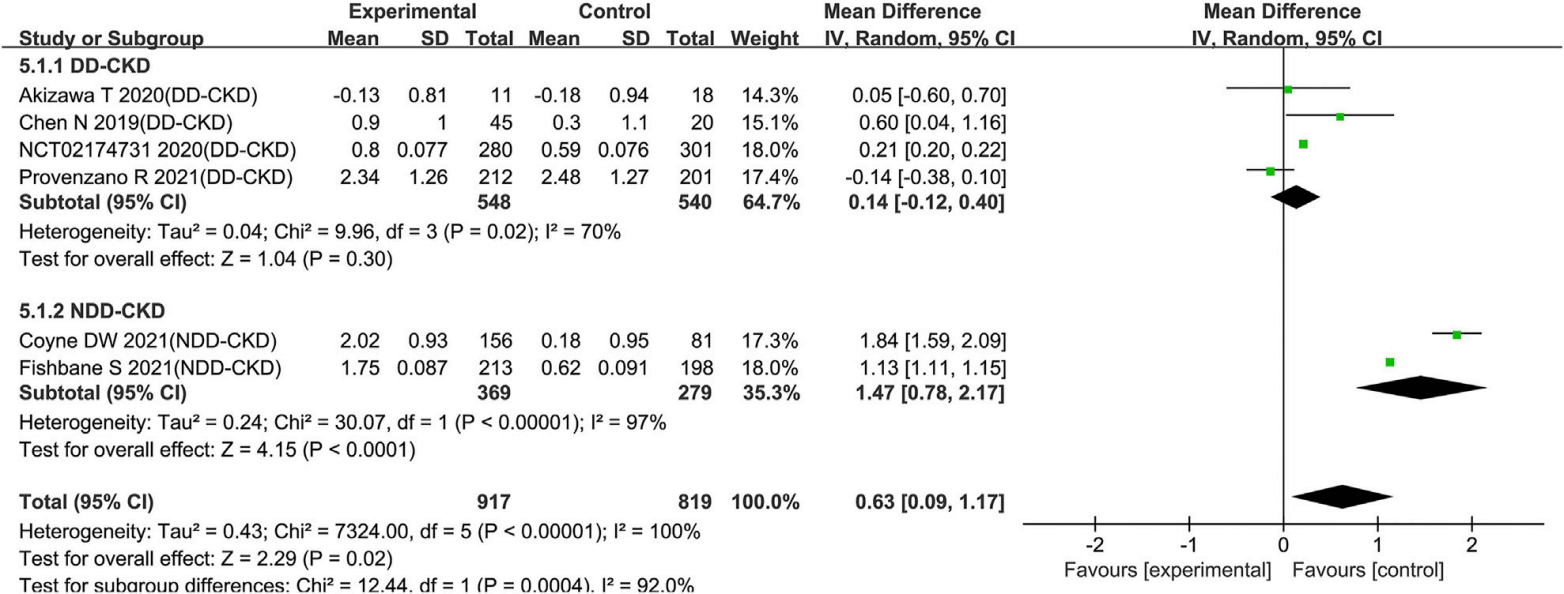
Effect of roxadustat compared with ESA or placebo on Hb level with elevated CRP levels in CKD patients. Notes: CKD, chronic kidney disease; DD-CKD, dialysis-dependent chronic kidney disease; NDD-CKD, non-dialysis-dependent chronic kidney disease; CRP, C-reactive protein.

Inflammation, which causes iron retention, increased hepcidin formation and impaired erythroid progenitor proliferation was believed as a major contributing factor to anemia (Macdougall et al., 2016; Weiss et al., 2019). In order to assess the influence of inflammation on the effect of roxadustat, we extracted Hb changes in subjects with baseline CRP > ULN from six trials (Chen et al., 2019a; Akizawa et al., 2019c; NCT02174731, 2020; Coyne et al., 2021; Fishbane et al., 2021; Provenzano et al., 2021) (four trials for DD-CKD patients, two trials for NDD-CKD patients). The pooled results showed that for patients with baseline CRP upper than ULN, there was a significant rise of Hb level with the use of roxadustat compared with ESAs and placebo (WMD: 0.63; 95% CI: 0.09–1.17; *p* = 0.02; with heterogeneity [*p* < 0.00001]; Figure 5 and Table 3). Subgroup analysis revealed that roxadustat significantly increase of Hb level compared with placebo in the NDD-CKD patients (WMD: 1.47; 95% CI: 0.78–2.17; *p* < 0.0001; with heterogeneity [*p* < 0.00001]; Figure 5 and Table 3) but not for DD-CKD patients.

#### Roxadustat Increased Serious Treatment-Emergent Adverse Events in Non-Dialysis-Dependent Chronic Kidney Disease Patients

**FIGURE 6:**
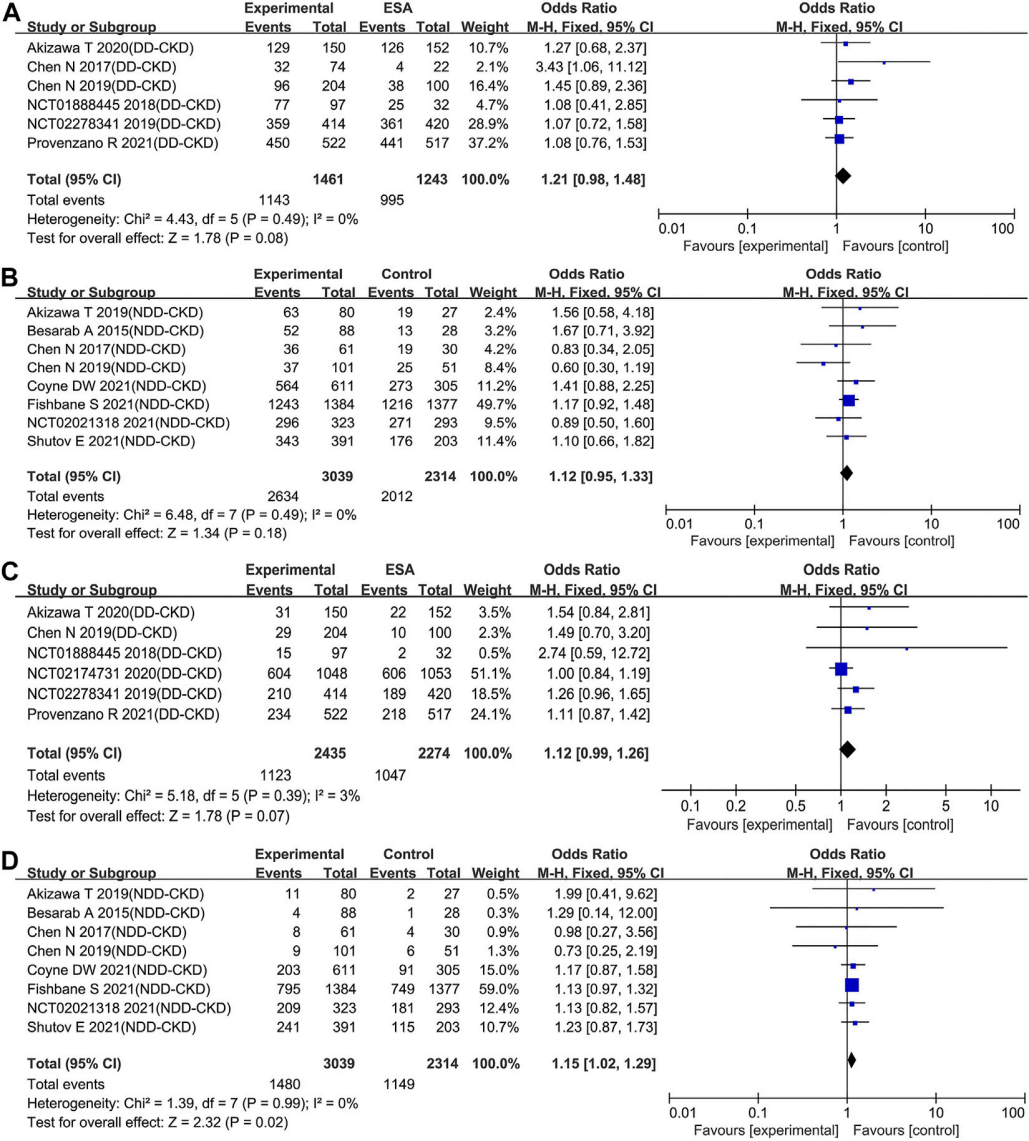
Effect of roxadustat compared with ESA or placebo on the incidence of TEAEs **(A, B)** and serious TEAEs **(C, D)** in CKD patients. Notes: CKD, chronic kidney disease; DD-CKD, dialysis-dependent chronic kidney disease; NDD-CKD, non-dialysis-dependent chronic kidney disease; TEAEs, treatment-emergent adverse events.

Nearly all of the included trials reported the incidence of TEAEs and serious TEAEs. Compared with ESAs or placebo, roxadustat did not increase the incidence of TEAEs neither in DD-CKD patient group (OR: 1.21; 95% CI: 0.98–1.48; *p* = 0.08; Figure 6A and Table 3) nor in NDD-CKD patient group (OR: 1.12; 95% CI: 0.95–1.33; *p* = 0.18; Figure 6B and Table 3). The same was true of cardiovascular events, including all-cause mortality, myocardial infarction, unstable angina, and congestive heart failure (online Supplementary Figure S2). Although there was no significant difference in the incidence of serious TEAEs between the use of roxadustat and the use of ESAs in DD-CKD patient group (OR: 1.12; 95% CI: 0.99–1.26; *p* = 0.07; Figure 6C and Table 3), roxadustat increased serious TEAEs in NDD-CKD patient group (OR: 1.15; 95% CI: 1.02–1.29; *p* = 0.02; Figure 6D and Table 3).

### Publication Bias and Sensitivity Analysis

Egger’s tests were utilized to estimate publication bias. No significant publication bias was found for primary-outcomes Hb levels (*p* = 0.481) and Hb response (*p* = 0.432). For secondary outcomes, no publication bias existed except for TIBC (*p* = 0.031) and Serious TEAE (*p* = 0.032) (online Supplementary Table S4). After the adjustment by trim and fill method, it indicated that publication bias has not affected the estimates of TIBC and serious TEAEs (online Supplementary Table S5). In the sensitivity analysis, kicking out the unpublished clinical trial data altered just one outcome. Comparing with ESAs, roxadustat failed to increase Hb levels in DD-CKD patient group (*p* = 0.05) when the unpublished clinical trials were excluded. The detailed results of meta-analysis were shown in the online Supplementary Figure S3–S6.

## Discussion

The results of our meta-analysis demonstrated that, compared with placebo, roxadustat increased Hb levels effectively and was associated with a remarkably higher rate of Hb response in NDD-CKD patients without increasing TEAEs. For DD-CKD patients, we found that the influence of roxadustat on Hb levels was inconsistent during the sensitivity analysis. While all trials were included in the analysis, it was found that roxadustat significantly increased Hb level (WMD: 0.14; 95% CI: 0.05–0.23; *p* = 0.001). In contrast, when three unpublished trials were kicked out, comparable Hb level was observed in both roxadustat and ESA group (WMD: 0.19; 95% CI: 0.00 to 0.37; *p* = 0.05) (online Supplementary Figure S3A). This was also consistent with the results of all phase 3 trials in DD-CKD patients, suggesting that roxadustat is non-inferiority to ESA. Different efficacy of roxadustat on changes of Hb levels was speculated to be caused by the following two reasons. Firstly, different control reagents were administrated in the trials, placebo in NDD-CKD group (except darbepoetin alfa was used in one trial (NCT02021318, 2021)) while ESAs in the DD-CKD group. Secondly, there was a gap between NDD-CKD group (9.0 g/dL) and DD-CKD group (10.0 g/dL) for baseline Hb levels. The heterogeneity of comparison reagents and baseline Hb levels contributed to the difference in efficacy of roxadustat.

It's worth noting that, serious TEAEs were increased after treatment of roxadustat in NDD-CKD group (OR: 1.15; 95% CI: 1.02–1.29; *p* = 0.02). The possible reason caused higher incidence of serious TEAEs of roxadustat is that, different comparators used for the control groups in DD-CKD patients and NDD-CKD patients. ESAs were administrated as comparator in DD-CKD patients, whereas the safety of ESAs to treat CKD-associated anemia has been questioned because of greater risks for death, serious adverse cardiovascular reactions and stroke (Singh et al., 2006; Pfeffer et al., 2009). Our results displayed that the incidence of serious TEAEs was similar in roxadustat and ESAs group (OR: 1.12; 95% CI: 0.99–1.26; *p* = 0.07), which could only indicate comparable safety concern of roxadustat and ESAs. In contrast, placebo was used in control group for NDD-CKD patients in most of the included trials except one in which darbepoetin alfa was used (NCT02021318, 2021). Using of placebo, a therapy without noteworthy drug-related adverse events, provided greater evidentiary power to allow valid assessment of safety of roxadustat (Fishbane et al., 2017; Fishbane et al., 2019). Our results indicated roxadustat increased serious TEAEs compared with placebo in NDD-CKD patients. However, recent annual conference of the American Society of Nephrology (ASN) summarized the data from multiple large global phase 3 trials of roxadustat and released the cardiovascular events data (Astrazeneca, 2019). For the NDD-CKD patients, there were no significant differences in the risks of major adverse cardiovascular events (MACEs), MACE^+^, and all-cause mortality between the roxadustat and placebo groups.

A significant decrease of hepcidin, TSAT and ferritin was observed in roxadustat group especially for NDD-CKD patients. And transferrin and TIBC were significantly increased after treatment of roxadustat. The package label of conventional ESA medicine indicates iron repletion and often, intravenous iron supplementation (Macdougall et al., 1996). HIF acts as an iron sensor, for example, HIF-2α upregulated iron absorption genes and increased serum iron (Peyssonnaux et al., 2007; Anderson et al., 2011). PHD inhibition by HIF-PHI (like roxadustat) stabilizes HIF, which further supresses hepcidin, improves intestinal iron absorption and increases in iron transport enzymes (Shah et al., 2009; Holdstock et al., 2016). Another advantage of HIF-PHI is activating several early response target genes, including the EPO gene and EPO receptor gene by transient inhibition of HIF prolyl hydroxylase (Suzuki et al., 2011). One of our included study Besarab et al. (2015) demonstrated endogenous EPO levels of subjects raised ∼4 h after dosing and peaked at ∼10 h on Day 1 and Day 29. Regrettably, the trials reported endogenous EPO levels after treatment of roxadustat is too scarce. More related studies are expected to facilitate evaluating the ability of roxadustat to increase endogenous EPO level.

Inflammation is one of the important cause anemia in CKD patients, which causes iron retention, increased hepcidin formation and impaired erythroid progenitor proliferation (Macdougall et al., 2016; Weiss et al., 2019). A substantial population of CKD patients does not respond to ESA therapy due to an underlying inflammatory state. Our results demonstrated that roxadustat increased the Hb level compared with placebo in NDD-CKD patients even with elevated CRP levels (WMD: 1.47; 95% CI: 0.78 to 2.17; *p* < 0.0001). But under the state of inflammation, roxadustat has no superiority in rising Hb levels in DD-CKD patients when ESAs were administrated as controls (WMD: 0.14; 95% CI: −0.12 to 0.40; *p* = 0.30). Notably, although the Hb levels were comparable between roxadustat group and ESAs-treated patients, higher dose of ESAs and more dose modifications were required for ESAs-treated patients under inflammation state.

The present study has several limitations. First, high heterogeneity exists in analysis results of some outcomes even after subgroup analysis. The heterogeneity maybe introduced by differences in the dose of administration, baseline Hb level, treatment duration, and iron supplementation. Second, the quality of evidence for the primary-outcomes is low or very low. Most of the included RCTs in the DD-CKD patients were open-label studies except two clinical trials from the Japanese population (NCT01888445, 2018; Akizawa et al., 2020a). The open-label studies would bring about high risks of performance and detection biases. Third, all the included trials were sponsored by the relevant pharmaceutical companies, which may have an adverse impact on the reliability of the results. For instance, roxadustat failed to increase Hb levels in DD-CKD patient group (*p* = 0.05) compared with ESAs when the unpublished clinical trials were excluded. Fourth, no existing post-marketing evaluation data was included. The results of two ongoing phase 4 clinical trials (NCT04059913 and NCT04134026) conducted in China will provide more comprehensive efficacy and safety profile of roxadustat.

## Conclusion

In summary, roxadustat can raise the Hb levels and regulate iron metabolism in both DD-CKD patients and NDD-CKD patients. For NDD-CKD patients, roxadustat was efficacious under inflammation state but increased serious TEAEs.

## Data Availability

The original contributions presented in the study are included in the article/Supplementary Material, further inquiries can be directed to the corresponding authors.
