# Supplementary material for: Safe and Effective Treatment for Anemic Patients With Chronic Kidney Disease: An Updated Systematic Review and Meta-Analysis on Roxadustat
Source: Front Pharmacol. 2021 Jul 2;12:658079. doi: 10.3389/fphar.2021.658079 (PMC8283176; doi:10.3389/fphar.2021.658079)
Supplement: Supplementary file 1 [file DataSheet1.docx]

Supplementary Material

# Supplementary Figures and Tables

## Supplementary Figures


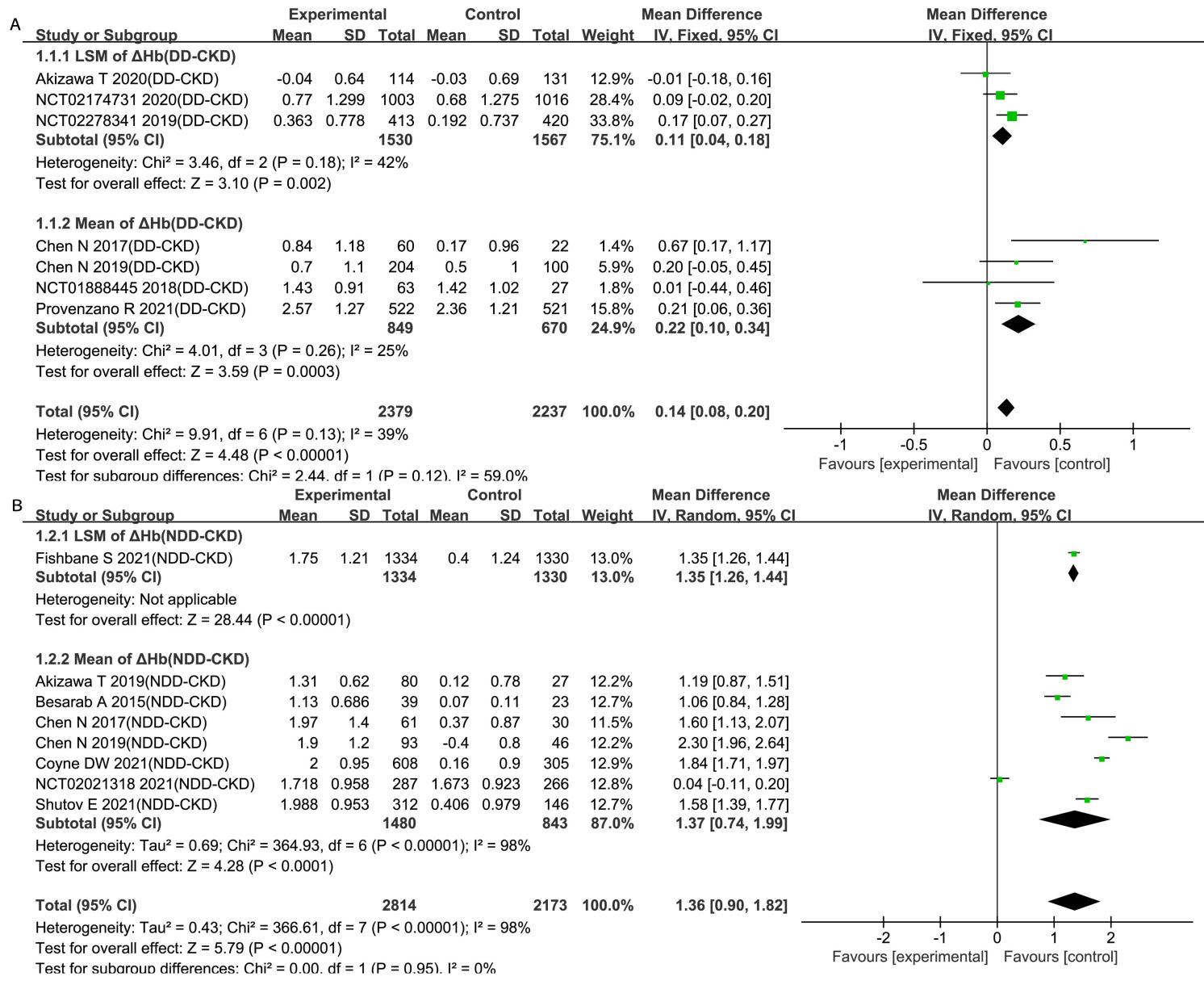


**Supplementary Figure 1.** Effect of roxadustat compared with ESA or placebo on Hb level in DD-CKD patients (A) and NDD-CKD patients (B)
Notes: CKD, chronic kidney disease; DD-CKD, dialysis-dependent chronic kidney disease; NDD-CKD, non-dialysis-dependent chronic kidney disease; LSM, least squares mean.


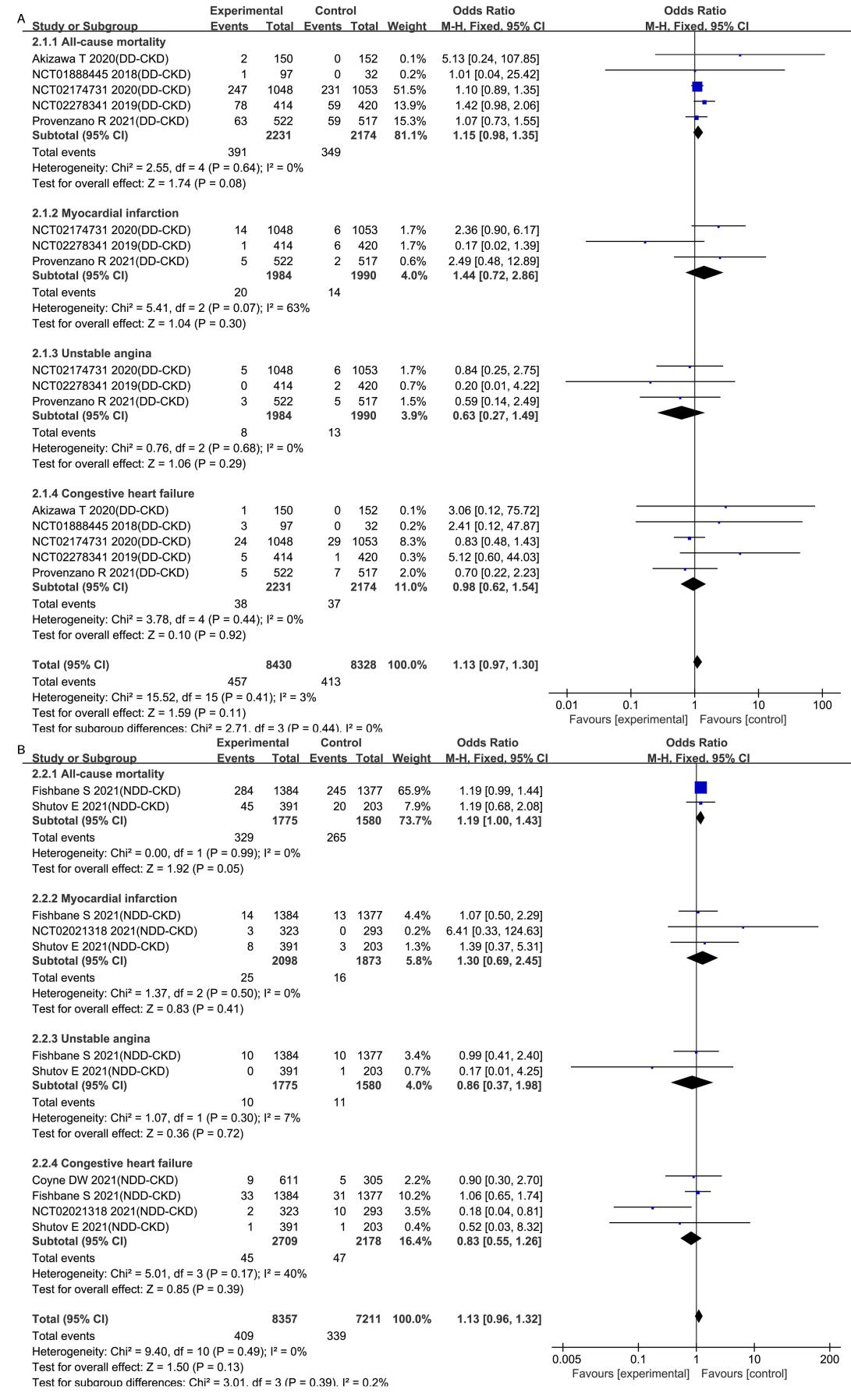


**Supplementary Figure 2.** Effect of roxadustat compared with ESA or placebo on cardiovascular events in DD-CKD patients (A) and NDD-CKD patients (B)

Notes: CKD, chronic kidney disease; DD-CKD, dialysis-dependent chronic kidney disease;NDD-CKD, non-dialysis-dependent chronic kidney disease.


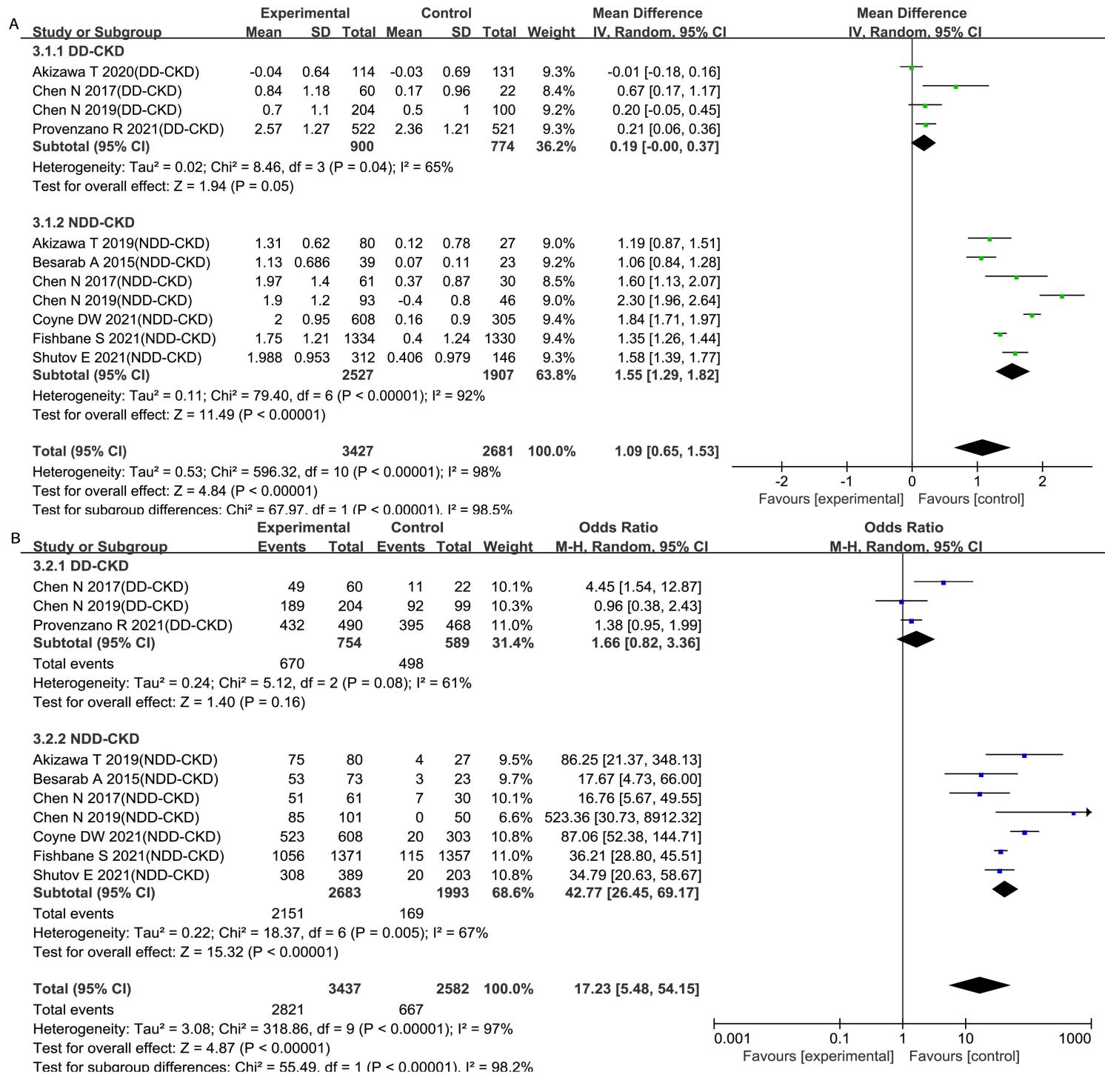


**Supplementary Figure 3.** Sensitivity analysis for Hb level (A) and Hb response (B)

Notes: Hb, hemoglobin; DD-CKD, dialysis-dependent chronic kidney disease; NDD-CKD, non-dialysis-dependent chronic kidney disease.


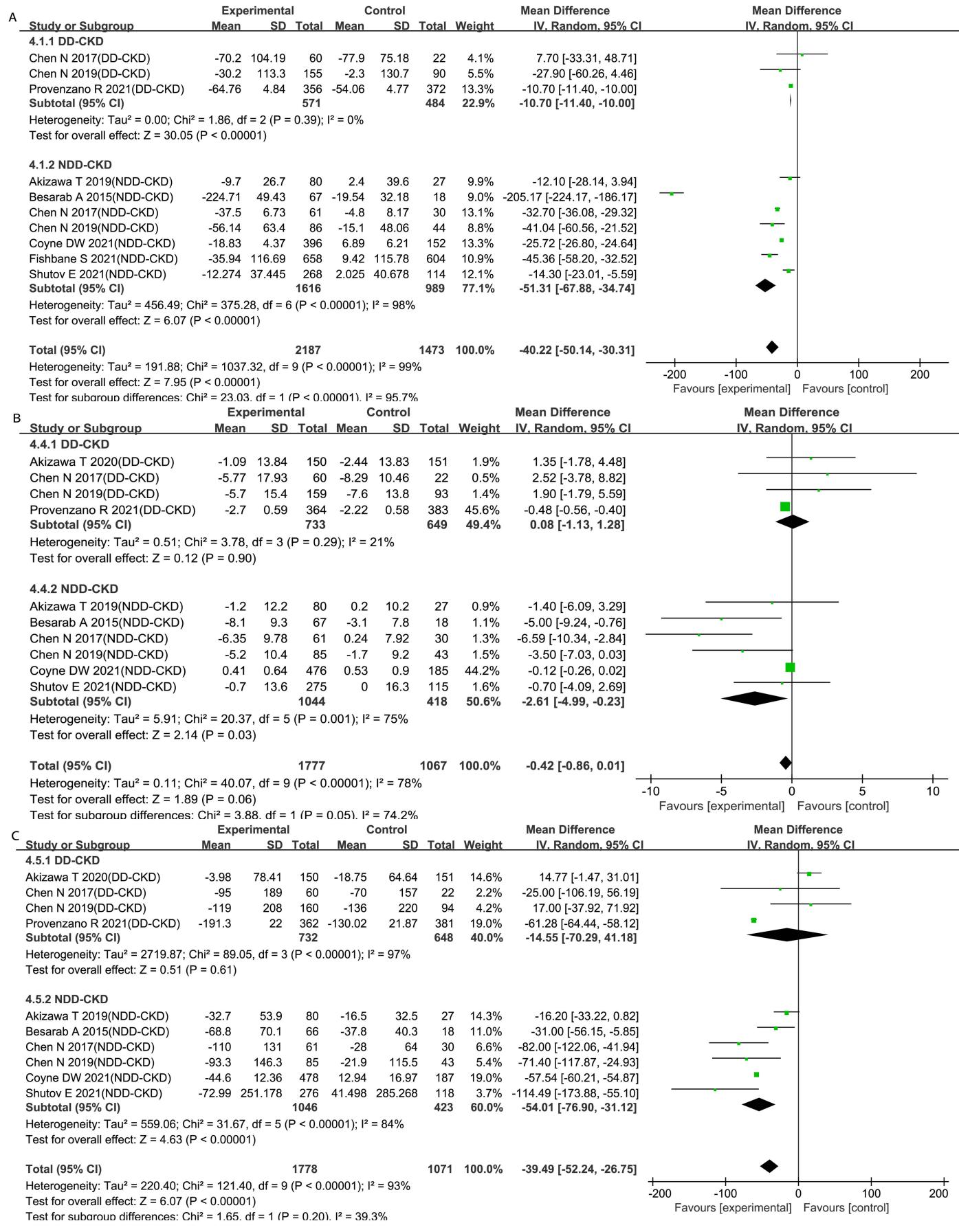


**Supplementary Figure 4.** Sensitivity analysis for iron utilization parameters. Iron utilization parameters included hepcidin (A), TSAT (B) and ferritin (C)

Notes: Hb, hemoglobin; DD-CKD, dialysis-dependent chronic kidney disease; NDD-CKD, non-dialysis-dependent chronic kidney disease.


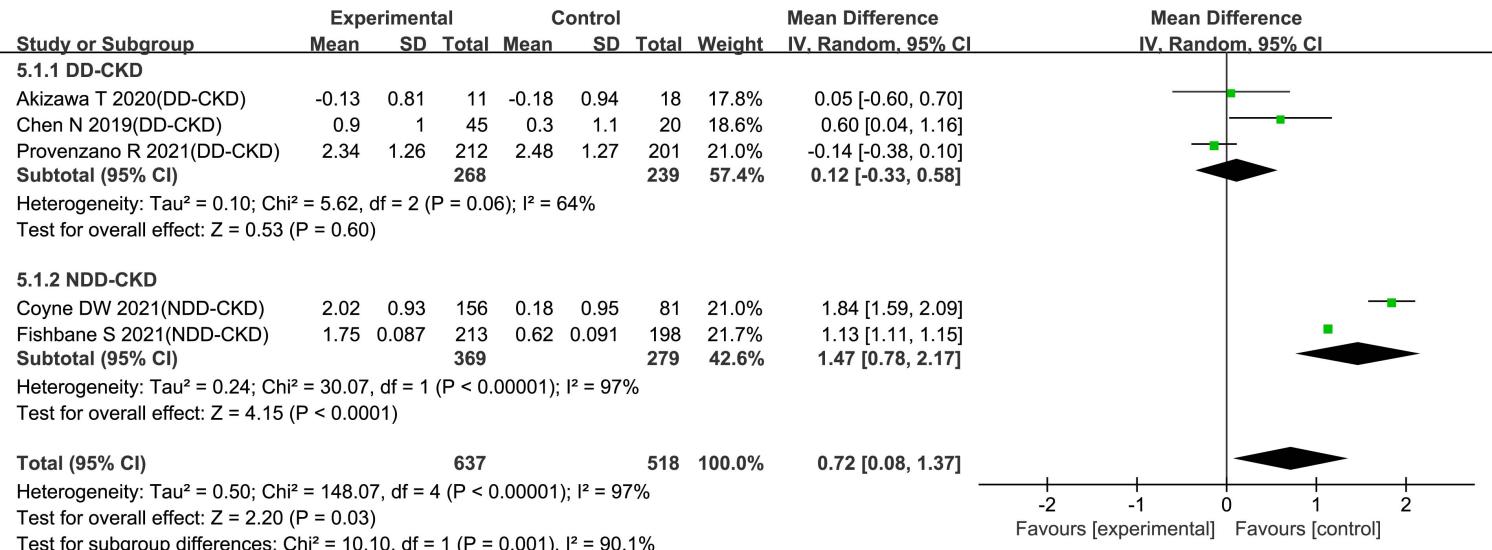


**Supplementary Figure 5.** Sensitivity analysis for Hb level with elevated CRP levels

Notes: Hb, hemoglobin; DD-CKD, dialysis-dependent chronic kidney disease; NDD-CKD, non-dialysis-dependent chronic kidney disease; CRP, C-reactive protein.


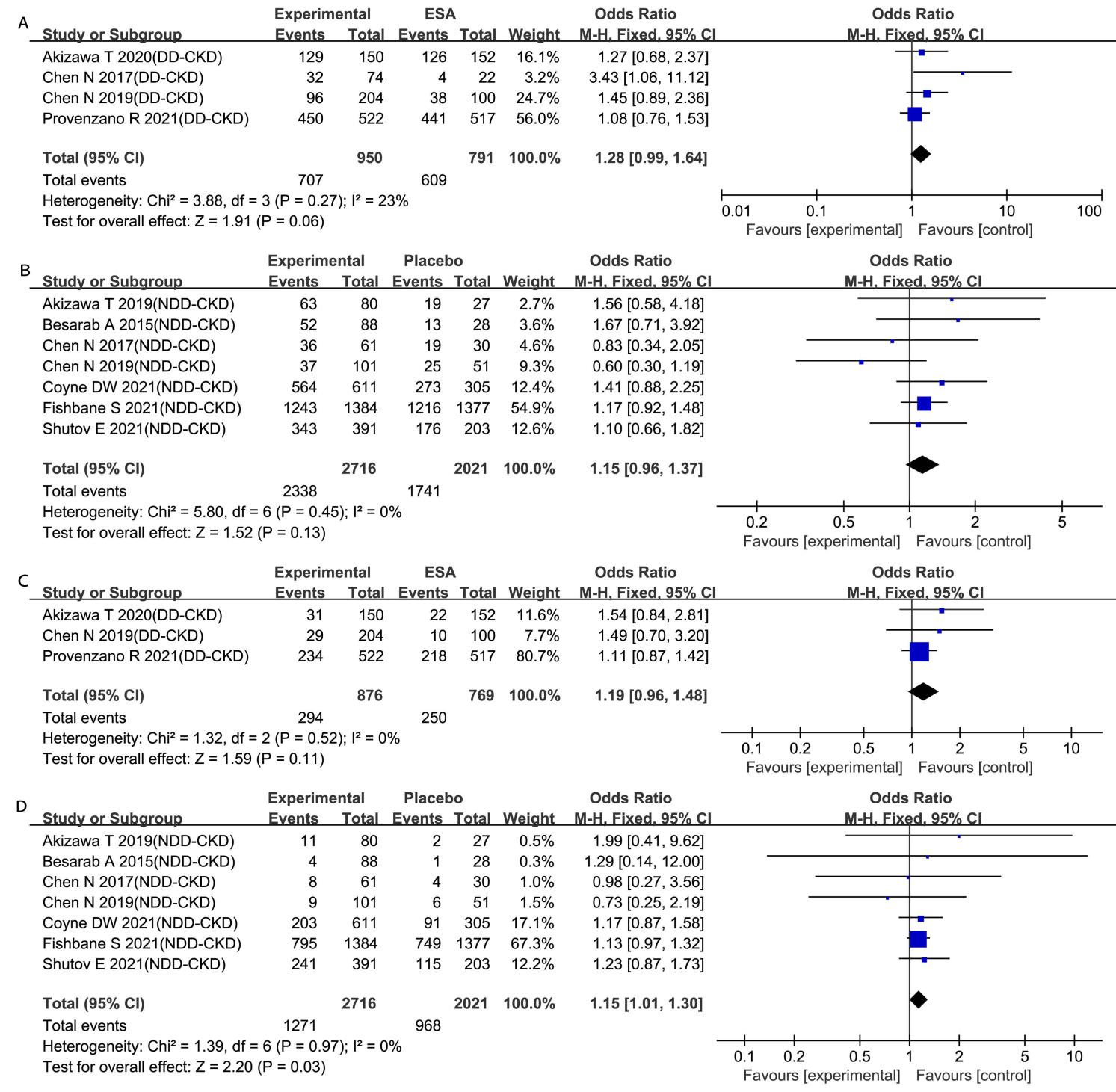


**Supplementary Figure 6.** Sensitivity analysis for the incidence of TEAEs (A, B) and serious TEAEs (C, D)

Notes: DD-CKD, dialysis-dependent chronic kidney disease; NDD-CKD, non-dialysis-dependent chronic kidney disease; TEAEs, treatment-emergent adverse events.

## Supplementary Tables

**Supplemental Table 1**. PRISMA checklist

| **Section/topic** | **#** | **Checklist item** | **Reported on page #** |
| --- | --- | --- | --- |
| **TITLE** | | |  |
| Title | 1 | Identify the report as a systematic review, meta-analysis, or both. | P.1 |
| **ABSTRACT** | | |  |
| Structured summary | 2 | Provide a structured summary including, as applicable: background; objectives; data sources; study eligibility criteria, participants, and interventions; study appraisal and synthesis methods; results; limitations; conclusions and implications of key findings; systematic review registration number. | P.1-2 |
| **INTRODUCTION** | | |  |
| Rationale | 3 | Describe the rationale for the review in the context of what is already known. | P.2-3 |
| Objectives | 4 | Provide an explicit statement of questions being addressed with reference to participants, interventions, comparisons, outcomes, and study design (PICOS). | P.3 |
| **METHODS** | | |  |
| Protocol and registration | 5 | Indicate if a review protocol exists, if and where it can be accessed (e.g., Web address), and, if available, provide registration information including registration number. | No |
| Eligibility criteria | 6 | Specify study characteristics (e.g., PICOS, length of follow-up) and report characteristics (e.g., years considered, language, publication status) used as criteria for eligibility, giving rationale. | P.4 |
| Information sources | 7 | Describe all information sources (e.g., databases with dates of coverage, contact with study authors to identify additional studies) in the search and date last searched. | P.4 |
| Search | 8 | Present full electronic search strategy for at least one database, including any limits used, such that it could be repeated. | **P.4**,  *Supplemental Table 2* |
| Study selection | 9 | State the process for selecting studies (i.e., screening, eligibility, included in systematic review, and, if applicable, included in the meta-analysis). | P.4 |
| Data collection process | 10 | Describe method of data extraction from reports (e.g., piloted forms, independently, in duplicate) and any processes for obtaining and confirming data from investigators. | P.5 |
| Data items | 11 | List and define all variables for which data were sought (e.g., PICOS, funding sources) and any assumptions and simplifications made. | P.5 |
| Risk of bias in individual studies | 12 | Describe methods used for assessing risk of bias of individual studies (including specification of whether this was done at the study or outcome level), and how this information is to be used in any data synthesis. | P.5 |
| Summary measures | 13 | State the principal summary measures (e.g., risk ratio, difference in means). | P.5 |
| Synthesis of results | 14 | Describe the methods of handling data and combining results of studies, if done, including measures of consistency (e.g., I^2^) for each meta-analysis. | P.5 |

**Supplemental Table 1**. Continued

| **Section/topic** | **#** | **Checklist item** | **Reported on page #** |
| --- | --- | --- | --- |
|  |  |  |  |
| Risk of bias across studies | 15 | Specify any assessment of risk of bias that may affect the cumulative evidence (e.g., publication bias, selective reporting within studies). | P.5 |
| Additional analyses | 16 | Describe methods of additional analyses (e.g., sensitivity or subgroup analyses, meta-regression), if done, indicating which were pre-specified. | P.5 |
| RESULTS | | |  |
| Study selection | 17 | Give numbers of studies screened, assessed for eligibility, and included in the review, with reasons for exclusions at each stage, ideally with a flow diagram. | P.5-6,  *Figure 1* |
| Study characteristics | 18 | For each study, present characteristics for which data were extracted (e.g., study size, PICOS, follow-up period) and provide the citations. | P.6,  *Table 1* |
| Risk of bias within studies | 19 | Present data on risk of bias of each study and, if available, any outcome level assessment (see item 12). | P.6,  *Table 2 and Figure 2* |
| Results of individual studies | 20 | For all outcomes considered (benefits or harms), present, for each study: (a) simple summary data for each intervention group (b) effect estimates and confidence intervals, ideally with a forest plot. | *Figure 3-6* |
| Synthesis of results | 21 | Present results of each meta-analysis done, including confidence intervals and measures of consistency. | P.7-8 |
| Risk of bias across studies | 22 | Present results of any assessment of risk of bias across studies (see Item 15). | P.9,  *Supplemental Table 4-5* |
| Additional analysis | 23 | Give results of additional analyses, if done (e.g., sensitivity or subgroup analyses, meta-regression [see Item 16]). | P.7-8,  *Supplemental Figure 3-6* |
| DISCUSSION | | |  |
| Summary of evidence | 24 | Summarize the main findings including the strength of evidence for each main outcome; consider their relevance to key groups (e.g., healthcare providers, users, and policy makers). | P.9,  *Supplemental Table 3* |
| Limitations | 25 | Discuss limitations at study and outcome level (e.g., risk of bias), and at review-level (e.g., incomplete retrieval of identified research, reporting bias). | P.11 |
| Conclusions | 26 | Provide a general interpretation of the results in the context of other evidence, and implications for future research. | P.11 |
| FUNDING | | |  |
| Funding | 27 | Describe sources of funding for the systematic review and other support (e.g., supply of data); role of funders for the systematic review. | P.11 |

**Supplemental Table 2**. Searching strategies

| Database | Searching strategies |
| --- | --- |
| PubMed | #1 ("Renal Insufficiency, Chronic"[Mesh]) OR ((((((((((((((((((((Chronic Renal Insufficiencies) ) OR (Renal Insufficiencies, Chronic)) OR (Chronic Renal Insufficiency)) OR (Kidney Insufficiency, Chronic)) OR (Chronic Kidney Insufficiency)) OR (Chronic Kidney Insufficiencies)) OR (Kidney Insufficiencies, Chronic)) OR (Chronic Kidney Diseases)) OR (Chronic Kidney Disease)) OR (Disease, Chronic Kidney)) OR (Diseases, Chronic Kidney)) OR (Kidney Disease, Chronic)) OR (Kidney Diseases, Chronic)) OR (Chronic Renal Diseases)) OR (Chronic Renal Disease)) OR (Disease, Chronic Renal)) OR (Diseases, Chronic Renal)) OR (Renal Disease, Chronic)) OR (Renal Diseases, Chronic))  #2 ((((roxadustat[All Fields])) OR (FG-4592)) OR (ASP1517)) OR (AZD9941)  #3 #1 and #2 |
| Web of Science | #1 TS=(Renal Insufficiency, Chronic OR Chronic Renal Insufficiencies OR Renal Insufficiencies, Chronic OR Chronic Renal Insufficiency OR Kidney Insufficiency, Chronic OR Chronic Kidney Insufficiency OR Chronic Kidney Insufficiencies OR Kidney Insufficiencies, Chronic OR Chronic Kidney Diseases OR Chronic Kidney Disease OR Disease, Chronic Kidney OR Diseases, Chronic Kidney OR Kidney Disease, Chronic OR Kidney Diseases, Chronic OR Chronic Renal Diseases OR Chronic Renal Disease OR Disease, Chronic Renal OR Diseases, Chronic Renal OR Renal Disease, Chronic OR Renal Diseases, Chronic)  #2 TS=(Roxadustat OR FG-4592 OR ASP1517 OR AZD9941)  #3 #1 AND #2 |
| Cochrane Library | (roxadustat) OR (FG-4592) OR (ASP1517) OR (AZD9941) |

**Supplemental Table 3.** The quality of the evidence for all outcomes are listed in the Summary of Findings (SoF) table

| **Outcomes** | **Subgroup** | **Illustrative comparative risks* (95% CI)** | | **Relative effect (95% CI)** | **No of Participants (studies)** | **Quality of the evidence (GRADE)** |
| --- | --- | --- | --- | --- | --- | --- |
|  |  | Assumed risk (**Control**) | Corresponding risk(**Roxadustat**) |  |  |  |
| **Hb** | **DD-CKD** |  | The mean hb - dd-ckd in the intervention groups was **0.14 higher** (0.05 to 0.23 higher) |  | 4616 (7 studies) | ⊕⊝⊝⊝ **very low**^1,2,3^ |
|  | **NDD-CKD** |  | The mean hb - ndd-ckd in the intervention groups was **1.36 higher** (0.9 to 1.82 higher) |  | 4987 (8 studies) | ⊕⊕⊝⊝ **low**^1,3^ |
| **Hb response** | **DD-CKD** | **Study population** | | **OR 1.27**  (0.86 to 1.87) | 2253 (5 studies) | ⊕⊝⊝⊝ **very low**^1,2,3^ |
|  |  | **828 per 1000** | **860 per 1000** (806 to 900) |  |  |  |
|  |  | **Moderate** | |  |  |  |
|  |  | **824 per 1000** | **856 per 1000** (801 to 897) |  |  |  |
|  | **DNN-CKD** | **Study population** | | **OR 30.29**  (11.55 to 79.42) | 5235 (8 studies) | ⊕⊕⊝⊝ **low**^1,3^ |
|  |  | **169 per 1000** | **860 per 1000** (701 to 942) |  |  |  |
|  |  | **Moderate** | |  |  |  |
|  |  | **115 per 1000** | **797 per 1000** (600 to 912) |  |  |  |
| **Hepcidin** | **DD-CKD** |  | The mean hepcidin - dd-ckd in the intervention groups was **11.49 lower** (14.58 to 8.41 lower) |  | 1722 (4 studies) | ⊕⊕⊝⊝ **low**^1,2,3^ |
|  | **NDD-CKD** |  | The mean hepcidin - ndd-ckd in the intervention groups was **51.31 lower** (67.88 to 34.74 lower) |  | 2605 (7 studies) | ⊕⊕⊝⊝ **low**^1,3^ |
| **Transferrin** | **DD-CKD** |  | The mean transferrin - dd-ckd in the intervention groups was **0.4 higher** (0.3 to 0.5 higher) |  | 637 (3 studies) | ⊕⊕⊝⊝ **low**^1,3^ |
|  | **NDD-CKD** |  | The mean transferrin - ndd-ckd in the intervention groups was **0.6 higher** (0.24 to 0.95 higher) |  | 326 (3 studies) | ⊕⊕⊕⊝ **moderate**^3^ |
| **TIBC** | **DD-CKD** |  | The mean tibc - dd-ckd in the intervention groups was **43.65 higher** (33.78 to 53.53 higher) |  | 1382 (4 studies) | ⊕⊕⊝⊝ **low**^1,3^ |
|  | **NDD-CKD** |  | The mean tibc - ndd-ckd in the intervention groups was **59.9 higher** (38.85 to 80.96 higher) |  | 1072 (5 studies) | ⊕⊕⊝⊝ **low**^1,3^ |
| **TSAT** | **DD-CKD** |  | The mean tsat - dd-ckd in the intervention groups was **0.35 lower** (1.06 lower to 0.36 higher) |  | 2048 (5 studies) | ⊕⊝⊝⊝ **very low**^1,2,3^ |
|  | **NDD-CKD** |  | The mean tsat - ndd-ckd in the intervention groups was **2.84 lower** (5.03 to 0.64 lower) |  | 1956 (7 studies) | ⊕⊕⊝⊝ **low**^1,3^ |
| **Ferritin** | **DD-CKD** |  | The mean ferritin - dd-ckd in the intervention groups was **33.64 lower** (83.39 lower to 16.11 higher) |  | 2063 (5 studies) | ⊕⊝⊝⊝ **very low**^1,2,3^ |
|  | **NDD-CKD** |  | The mean ferritin - ndd-ckd in the intervention groups was **54.01 lower** (76.9 to 31.12 lower) |  | 1469 (6 studies) | ⊕⊕⊝⊝ **low**^1,3^ |
| **CRP** | **DD-CKD** |  | The mean crp - dd-ckd in the intervention groups was **0.14 higher** (0.12 lower to 0.4 higher) |  | 1089 (4 studies) | ⊕⊝⊝⊝ **very low**^1,2,3^ |
|  | **NDD-CKD** |  | The mean crp - ndd-ckd in the intervention groups was **1.47 higher** (0.78 to 2.17 higher) |  | 648 (2 studies) | ⊕⊕⊕⊝ **moderate**^3^ |
| **TEAE** | **DD-CKD** | **Study population** | | **OR 1.21**  (0.98 to 1.48) | 2704 (6 studies) | ⊕⊕⊝⊝ **low**^1,3^ |
|  |  | **800 per 1000** | **829 per 1000** (797 to 856) |  |  |  |
|  |  | **Moderate** | |  |  |  |
|  |  | **805 per 1000** | **833 per 1000** (802 to 859) |  |  |  |
|  | **NDD-CKD** | **Study population** | | **OR 1.12**  (0.95 to 1.33) | 5353 (8 studies) | ⊕⊝⊝⊝ **very low**^1,2,3^ |
|  |  | **869 per 1000** | **882 per 1000** (864 to 899) |  |  |  |
|  |  | **Moderate** | |  |  |  |
|  |  | **785 per 1000** | **804 per 1000** (776 to 829) |  |  |  |
| **Serious TEAE** | **DD-CKD** | **Study population** | | **OR 1.12**  (0.99 to 1.26) | 4709 (6 studies) | ⊕⊕⊝⊝ **low**^1,3^ |
|  |  | **460 per 1000** | **489 per 1000** (458 to 518) |  |  |  |
|  |  | **Moderate** | |  |  |  |
|  |  | **283 per 1000** | **307 per 1000** (281 to 332) |  |  |  |
|  | **NDD-CKD** | **Study population** | | **OR 1.15**  (1.02 to 1.29) | 5353 (8 studies) | ⊕⊝⊝⊝ **very low**^1,2,3^ |
|  |  | **497 per 1000** | **531 per 1000** (501 to 560) |  |  |  |
|  |  | **Moderate** | |  |  |  |
|  |  | **216 per 1000** | **241 per 1000** (219 to 262) |  |  |  |
| GRADE Working Group grades of evidence: **High quality:** Further research is very unlikely to change our confidence in the estimate of effect. **Moderate quality:** Further research is likely to have an important impact on our confidence in the estimate of effect and may change the estimate. **Low quality:** Further research is very likely to have an important impact on our confidence in the estimate of effect and is likely to change the estimate. **Very low quality:** We are very uncertain about the estimate. | | | | | | |
| ^1^ Some studies that did not implement blinding may introduce a risk of bias, so the quality of evidence was reduced by one level.  ^2^ The results of different studies were inconsistent, so the quality of evidence was reduced by one level.  ^3^ All studies were sponsored by pharmaceutical companies, so the quality of evidence was reduced by one level. | | | | | | |

**Supplemental Table 4.** Publication bias

| **Outcomes** | **Publication bias** |
| --- | --- |
| Hb |  |
| Hb response |  |
| Hepcidin |  |
| Transferrin |  |
| TIBC |  |
| TSAT |  |
| Ferritin |  |
| CRP |  |
| TEAE |  |
| Serious TEAE |  |

**Supplemental Table 5.** Adjusted publication bias

| **Outcomes** | **Adjusted publication bias** |
| --- | --- |
| TIBC |  |
| Serious TEAE |  |
